# Multi-omics profiling identifies a putative *Akkermansia*–HK3–lactate immunometabolic link associated with JPZS treatment in lupus nephritis

**DOI:** 10.3389/fimmu.2026.1873518

**Published:** 2026-10-22

**Authors:** Ming Li, Lijun Pang, Yunfei Li, Shuangshuang Shang, Chuanbing Huang

**Affiliations:** 1 Department of Rheumatology, The First Affiliated Hospital of Anhui University of Chinese Medicine, Hefei, Anhui, China; 2 Center for Xin’an Medicine and Modernization of Traditional Chinese Medicine, Institute of Health and Medicine, Hefei, Anhui, China

**Keywords:** akkermansia, gut microbiota, gut–kidney axis, herbal medicine, hexokinase 3, immunometabolism, lupus nephritis, multi-omics

## Abstract

**Background:**

Lupus nephritis (LN) is associated with aberrant glycolytic reprogramming in renal immune cells, yet its relationship with gut microbial alterations remains poorly defined. Hexokinase 3 (HK3) has emerged as a candidate metabolic node in myeloid activation, while the relationship between gut *Akkermansia* abundance and renal HK3-associated metabolic changes in LN remains unclear. We used Qihuang Jianpi Zishen decoction (JPZS), an herbal formula used as an adjunct in clinical practice, as an experimental probe to investigate this putative gut–immunometabolic connection through integrated multi-omics.

**Methods:**

Female MRL/lpr mice (*n* = 6 per group) received JPZS (6.305, 12.61, or 25.22 g/kg/day) or vehicle for 8 weeks, with C57BL/6 mice serving as a healthy reference group. Renal function, autoantibodies, histopathology, and glomerular IgG deposition were assessed across treatment groups. Integrated multi-omics profiling—fecal 16S rRNA sequencing, untargeted plasma metabolomics, and renal RNA sequencing—was performed in the matched control, model, and high-dose JPZS groups. Renal lactate and pyruvate were quantified, and HK3 and Ldhal6b mRNA and their corresponding proteins were evaluated by qPCR and western blotting. HK3/F4/80 dual immunofluorescence was additionally performed to examine the spatial localization of HK3 relative to renal macrophages.

**Results:**

JPZS reduced proteinuria, serum creatinine, glomerular IgG deposition, and renal histopathological injury in MRL/lpr mice. Gut microbiome profiling showed marked enrichment of *Akkermansia* and altered community composition, without significant recovery of Chao1 or Shannon diversity, accompanied by reductions in *Klebsiella* and *Enterococcus*. Plasma metabolomics revealed remodeling of amino-acid and central carbon metabolism, while renal transcriptomics identified immune-related differences and an exploratory glycolysis/gluconeogenesis enrichment signal. JPZS reduced renal lactate accumulation and the lactate/pyruvate ratio and decreased renal HK3 expression at both the mRNA and protein levels. Dual immunofluorescence showed increased HK3 and F4/80 fluorescence in model kidneys, with overlapping signals evident in merged images; both signals were lower following JPZS-H treatment. Cross-layer correlation analyses linked gut genera, circulating metabolites, and renal gene expression; targeted assays separately characterized renal lactate/pyruvate status.

**Conclusion:**

JPZS ameliorated renal injury while inducing coordinated changes in the gut microbiome, circulating metabolome, and renal immunometabolic profile in MRL/lpr mice. Integrated multi-omics and targeted validation identified a putative *Akkermansia*–HK3–lactate immunometabolic link associated with JPZS treatment. These findings provide a hypothesis-generating framework for gut–kidney immunometabolic crosstalk in LN and warrant future causal validation through microbiota- and HK3-targeted approaches.

## Introduction

Lupus nephritis (LN), affecting 40%–60% of patients with systemic lupus erythematosus (SLE), remains a leading cause of chronic kidney disease, end-stage kidney disease, and premature death despite advances in immunosuppressive therapy (1–3). Current standard-of-care regimens—glucocorticoids combined with mycophenolate mofetil or cyclophosphamide, with or without calcineurin inhibitors—achieve incomplete remission in a substantial proportion of patients. Even with newer add-on therapies, complete renal response was achieved in 30% of patients receiving belimumab at 104 weeks and 41% of those receiving voclosporin at 52 weeks (4–6). These persistent limitations underscore the need to identify pathogenic axes beyond classical autoantibody production and immune-complex deposition (7). Dysregulated immunometabolism has emerged as one such axis: in the lupus kidney, infiltrating monocytes and macrophages adopt an HIF-1α-driven glycolytic program that amplifies IL-1β production, promotes leukocyte recruitment, and sustains immune-complex-mediated tissue injury; inhibiting this program attenuates renal inflammation *in vivo*, establishing glycolytic reprogramming in renal myeloid cells as a therapeutically relevant target (8, 9). Within the glycolytic machinery, hexokinase 3 (HK3) is of particular interest: enriched in granulocytes and myeloid lineages, HK3 supports cell survival and shapes inflammatory crosstalk through both glycolytic and non-glycolytic mechanisms (10, 11). However, the expression pattern and cellular localization of HK3 in lupus nephritis remain poorly defined, leaving an important gap between the broader concept of immunometabolic dysregulation and the cellular context of HK3-associated metabolic alterations in the lupus kidney.

Alongside immunometabolic dysregulation, a disturbed gut–kidney axis contributes to LN pathogenesis. Patients with SLE and LN exhibit reduced microbial diversity, expansion of pathobionts, and altered microbially derived metabolite profiles; experimental dysbiosis accelerates renal inflammation and immune-complex glomerulonephritis in lupus-prone mice, while uremia and renal injury reciprocally reshape the intestinal ecosystem (12–14). Within this context, *Akkermansia muciniphila* has emerged as an immunometabolically active commensal of particular relevance: reduced abundance has been reported in lupus models, and supplementation in MRL/lpr mice lessens systemic inflammation, reinforces gut barrier integrity, and improves renal function (15–17). Beyond compositional effects, *A. muciniphila* modulates Toll-like receptor signaling, promotes regulatory T-cell responses, and reprograms macrophage polarization—properties that position it as a potential bridge between gut ecology and renal immune tone (18). Diet-derived polyphenols, polysaccharides, and phytochemicals selectively enrich *Akkermansia* and reshape microbial carbohydrate metabolism, providing a plausible route by which complex plant-derived interventions could influence the gut–kidney immunometabolic axis (19, 20). However, whether changes in *Akkermansia* abundance are associated with renal HK3-related metabolic alterations in lupus nephritis—and whether these coordinated changes are modulated by therapeutic intervention—remains unknown.

Qihuang Jianpi Zishen decoction (JPZS) is a clinically used, spleen–kidney-tonifying herbal formula comprising *Astragalus mongholicus* Bunge, *Rehmannia glutinosa*, *Cuscuta chinensis*, *Dioscorea oppositifolia*, *Poria cocos*, *Atractylodes macrocephala*, *Rubus chingii*, and *Rosa laevigata* (21–23). In MRL/lpr mice, JPZS reduces proteinuria, serum creatinine, and glomerular immune-complex injury, and modulates macrophage polarization, B-cell differentiation, and Treg/Th17 balance through multiple signaling pathways (21, 22, 24). Clinically, JPZS added to standard therapy improves proteinuria and composite renal outcomes in LN patients with spleen–kidney deficiency (25). Despite these multilevel effects, JPZS has not been studied with integrated 16S rRNA sequencing, plasma metabolomics, and renal transcriptomics, and it remains unknown whether JPZS-associated changes in gut *Akkermansia* are coordinated with alterations in renal HK3-associated metabolism.

We therefore used JPZS as an experimental probe in the MRL/lpr model to investigate whether treatment-associated alterations in the gut microbiota—particularly changes in *Akkermansia* abundance—are coordinated with renal HK3-associated metabolic alterations and intrarenal lactate accumulation along the gut–kidney axis. To this end, we integrated fecal 16S rRNA sequencing, untargeted plasma metabolomics, and renal RNA sequencing with conventional immunological and renal endpoints, followed by targeted validation of HK3, Ldhal6b, and renal lactate metabolism. HK3/F4/80 dual-immunofluorescence analysis was additionally performed to examine the spatial localization of renal HK3 relative to macrophages. Our goal was to determine whether these multi-omic changes characterize a putative *Akkermansia*–HK3–lactate association and to provide a hypothesis-generating framework for subsequent causal and translational investigation in immune-mediated kidney disease.

## Materials and methods

### Animals and study design

Female MRL/lpr mice (*n* = 30; 6 weeks old; 20 ± 2 g) and female C57BL/6 mice (*n* = 6; 6 weeks old; 20 ± 2 g) were purchased from Shanghai Slack Laboratory Animal Co., Ltd. [license no. SCXK(HU)2022-0004] and housed in a specific pathogen-free facility at 23 °C–25°C, 50%–70% relative humidity, under a 12-h light–dark cycle with free access to standard chow and water. After 1 week of acclimation, MRL/lpr mice were randomly assigned to five groups (*n* = 6 per group): lupus model (vehicle), JPZS low-dose (JPZS-L, 6.305 g/kg/day), JPZS medium-dose (JPZS-M, 12.61 g/kg/day), JPZS high-dose (JPZS-H, 25.22 g/kg/day), and prednisone acetate-positive control (PED, 4.55 mg/kg/day); C57BL/6 mice served as a healthy reference group. Animal doses were derived from the clinical adult daily dose using a human-to-mouse body surface area conversion factor of 9.01. All treatments were administered by oral gavage once daily for 8 weeks; prednisone acetate tablets (Tianjin Tianyao Pharmaceutical Co., Ltd., approval no. H12020689) were pulverized and suspended in 0.9% NaCl immediately before use, and control and model mice received equal volumes of vehicle.

All six groups were evaluated for systemic autoimmunity, renal function, and histopathology; for multi-omics profiling (16S rRNA sequencing, plasma metabolomics, and renal RNA sequencing) and targeted validation of glycolytic endpoints, three matched groups were analyzed—control, model, and JPZS-H (*n* = 6 per group)—to enable integrative cross-platform analyses. PED served as a pharmacological positive control for conventional disease endpoints and selected metabolic measurements but was not included in multi-omics integration because omics data were not generated for this group. Histopathological assessment, immunofluorescence quantification, ImageJ-based image analysis, and western blot densitometry were performed blinded to group allocation. All procedures were approved by the Institutional Animal Care and Use Committee of Anhui University of Chinese Medicine (approval no. AHUCM-mouse-2022130) and conducted in accordance with institutional guidelines for laboratory animal care and use.

### JPZS formula composition and extract preparation

JPZS is a standardized hospital-made granule formulation dispensed at our institution for patients with SLE and lupus nephritis. For the present animal study, JPZS was prepared as a water decoction using the same crude-herb composition and an equivalent crude-drug dose as the clinical granules. The formula comprised eight ingredients: *A. mongholicus* Bunge (Huangqi), *C. chinensis* Lam. (Tusizi), *A. macrocephala* Koidz. (Baizhu), *R. glutinosa* Steud. (Dihuang), *D. oppositifolia* L. (Shanyao), *P. cocos* F.A. Wolf (Fuling), *R. chingii* Hu (Fupenzi), and *R. laevigata* Michx. (Jinyingzi); botanical names were verified against World Flora Online and the fungal name against Index Fungorum/MycoBank (both accessed October 2025). All crude materials were sourced from the pharmacy of The First Affiliated Hospital of Anhui University of Chinese Medicine and authenticated by Zhang Mingsheng (Chief TCM Pharmacist) and Li Lihua (Chief Pharmacist) of the same institution. Crude herbs were soaked in 10 volumes of water for 1 h, decocted twice for 1.5 h each, combined, filtered, and concentrated by rotary evaporation to yield a water extract at 2.522 g/mL, which was stored at 4°C until use. The formula composition and batch were identical to those used in our previous study (22), and the chemical profile of the preparation is provided in **Supplementary Figure S1** and **Supplementary Table S1**. Targeted LC–MS/MS profiling identified 20 representative constituents, including astragaloside A, formononetin, quercetin, kaempferol, rutin, gallic acid, ellagic acid, atractylenolide III, dioscin, and pachymic acid, providing chemical characterization information for the JPZS preparation used in this study.

### Sample collection

After a 12-h fast, mice were anesthetized with 1% pentobarbital sodium (50 mg/kg, intraperitoneally), and blood was collected from the retro-orbital plexus into serum-separator tubes; serum was obtained by centrifugation at 3,000×*g* for 15 min at 4 °C and stored at −80 °C. For metabolomics, blood was collected separately into EDTA-containing tubes and centrifuged at 1,500–2,000×*g* for 10–15 min at 4°C within 30 min of collection. Plasma was aliquoted and stored at −80°C within 1 h of blood collection. Urine was collected in metabolic cages during the 12 h preceding euthanasia. Fecal pellets were collected immediately after defecation, snap-frozen in liquid nitrogen, and stored at −80°C for microbiome analysis. Following terminal blood collection, mice were euthanized by an overdose of pentobarbital sodium (≥150 mg/kg, intraperitoneally). Kidneys were excised and divided: one portion was fixed in 4% paraformaldehyde for histological and immunofluorescence analyses, and the remaining portions were snap-frozen at −80°C for transcriptomic and biochemical assays.

### Renal histology and immunofluorescence

Kidney tissue was fixed in 4% buffered paraformaldehyde for 48 h, embedded in paraffin, and sectioned at 3 µm. Sections were stained with H&E, PAS, and Masson’s trichrome, and brightfield images were acquired on an Olympus CX41 microscope. For glomerular IgG immunofluorescence, paraffin sections underwent citrate-based antigen retrieval (pH 6.0; ZSGB-BIO (Beijing, China), ZLI-9065), incubation with a rabbit anti-IgG antibody (1:500; Proteintech (Wuhan, China), 30000-0-AP), and TSA-based fluorescence visualization (green channel; Zhongkang Biotech (Hefei, China), D1002plus-01RM) with DAPI nuclear counterstaining. Images were acquired on a Pannoramic MIDI digital slide scanner (3DHISTECH, Hungary), and mean fluorescence intensity of IgG deposits was quantified from five glomeruli per section using ImageJ by a blinded observer.

To further assess the spatial localization of renal HK3 relative to macrophages, dual immunofluorescence staining for HK3 and the macrophage marker F4/80 was performed on kidney sections from the control, model, and JPZS-H groups (*n* = 3 independent mice per group). Sections were subjected to antigen retrieval and sequentially incubated with antibodies against HK3 (Proteintech, 13333-1-AP, rabbit, 1:100) and F4/80 (Santa Cruz Biotechnology (Dallas, TX, USA), sc-377009, mouse, 1:300), followed by HRP-conjugated secondary antibodies and TSA-based fluorescent detection using a multiplex immunofluorescence kit (Zhongkang Biotech, D1003plus-03RM). Nuclei were counterstained with DAPI. Images were acquired under identical settings using a Pannoramic MIDI digital slide scanner (3DHISTECH, Hungary). Mean optical densities of HK3 and F4/80 fluorescence were quantified separately using ImageJ, with the individual animal as the statistical unit.

### Serum and urine biochemistry

Serum antinuclear antibody (ANA) levels were quantified using a commercial sandwich ELISA kit (Shanghai Enzyme-linked Biotechnology Co., Ltd. (Shanghai, China), m1E1634) at 450 nm according to the manufacturer’s protocol. Serum anti-double-stranded DNA (anti-dsDNA) antibody concentrations were measured using a sandwich ELISA kit (Wuhan Jiyinmei Biotechnology (Wuhan, China), JYM1061Mo) at 450 nm according to the manufacturer’s protocol. Serum creatinine (S-Cr) was determined by the sarcosine oxidase colorimetric method (Nanjing Jiancheng Bioengineering Institute (Nanjing, China), C011-2-1) at 546 nm. Blood urea nitrogen (BUN), assessed as an ancillary positive-control renal-function endpoint, was measured using a urease-based colorimetric kit (Nanjing Jiancheng Bioengineering Institute (Nanjing, China), C013-2-1) at 640 nm according to the manufacturer’s instructions. Urinary total protein was quantified by the Coomassie Brilliant Blue method (Nanjing Jiancheng, C035-2-1) at 595 nm, and urinary creatinine was measured using the sarcosine oxidase method (Nanjing Jiancheng Bioengineering Institute, C011-2-1) at 546 nm after 10-fold dilution with saline; concentrations were corrected for the dilution factor. The urine protein-to-creatinine ratio (UPCR, g/g) was calculated from matched urine specimens collected over 12 h in metabolic cages. The kidney index was calculated as kidney weight (mg) divided by body weight (g).

### 16S rRNA gene sequencing and bioinformatics

Fecal DNA was extracted using the QIAamp DNA Stool Mini Kit (Qiagen, Germany). The V3–V4 hypervariable region of the 16S rRNA gene was amplified using primers 338F (5′-CCTACGGGNGGCWGCAG-3′) and 806R (5′-GACTACHVGGGTATCTAATCC-3′). Triplicate PCR products per sample were pooled, indexed, and purified with AMPure XP beads (Beckman Coulter, USA); library quality was verified by Qubit 2.0 fluorometer and Bioanalyzer 2100 before paired-end sequencing (2 × 250 bp) on an Illumina NovaSeq 6000 platform. Raw reads were processed in QIIME2: adapter trimming was performed with cutadapt, followed by denoising, paired-end merging, and chimera removal with DADA2, yielding 65,681–71,664 non-chimeric reads per sample (mean approximately 68,608 reads). For the three-group comparison, samples were rarefied to 65,681 reads per sample before downstream diversity analyses. Amplicon sequence variants (ASVs) were taxonomically classified against the SILVA 138.2 database at a confidence threshold of 0.8. Alpha diversity indices (Chao1, Shannon, and Simpson) and rarefaction curves were calculated in QIIME2. Beta diversity was assessed using Bray–Curtis dissimilarity and unweighted UniFrac distance, visualized by principal coordinates analysis (PCoA) and non-metric multidimensional scaling (NMDS), and tested by PERMANOVA with 9,999 permutations using the vegan R package. ANOSIM based on Bray–Curtis dissimilarity was additionally used to assess between-group versus within-group community dissimilarity. Differentially abundant taxa were identified by LEfSe (*p* < 0.05, LDA score > 2.0). Predicted functional profiles were inferred using PICRUSt2(v2.3.0) and mapped to KEGG pathways; as these predictions are based on marker-gene data rather than direct metagenomic sequencing, they are considered exploratory and hypothesis-generating only.

### Untargeted plasma metabolomics

EDTA-anticoagulated plasma samples were thawed at 4°C and vortex-mixed for 1 min. An aliquot of each sample was mixed with 400 μL methanol and centrifuged at 12,000 rpm for 10 min at 4°C. The resulting supernatant was concentrated to dryness and reconstituted in 150 μL of 80% methanol containing 4 ppm 2-chloro-L-phenylalanine, followed by filtration through a 0.22-μm membrane. Plasma metabolites were profiled on a Vanquish UHPLC system coupled to an Orbitrap Exploris 120 mass spectrometer (Thermo Fisher Scientific, USA) operated in both positive and negative electrospray ionization modes. Chromatographic separation was performed on an ACQUITY UPLC HSS T3 column (1.8 μm, 2.1 × 100 mm; Waters (Milford, MA, USA)) at 40°C, with a flow rate of 0.30 mL/min and an injection volume of 2 μL. For positive-ion acquisition, mobile phases A and B were 0.1% formic acid in water and 0.1% formic acid in acetonitrile, respectively; for negative-ion acquisition, mobile phases A and B were 5 mM ammonium formate in water and acetonitrile, respectively. The gradient was 10% B at 0–1 min, 10%–98% B at 1–5 min, 98% B at 5–6.5 min, 98%–10% B at 6.5–6.6 min, and 10% B at 6.6–8 min. Full-scan data were acquired over *m*/*z* 100–1,000 at a resolution of 60,000, with data-dependent MS/MS acquired at a resolution of 15,000 using 30% HCD collision energy. Pooled quality-control (QC) samples prepared by combining equal aliquots of all plasma samples were injected every 10 samples throughout the run to monitor instrument stability and signal drift.

Raw files were converted to mzXML format using MSConvert (v3.0.8789) and processed in XCMS (v3.12.0) for peak detection, retention-time alignment, and feature extraction using the centWave algorithm (ppm = 15, peakwidth = 5–30 s, bw = 2, mzwid = 0.015, and mzdiff = 0.01). Peak intensities were normalized to the total peak area. Metabolite annotation was performed against HMDB, MassBank, METLIN, MoNA, mzVault, KEGG, and an in-house spectral library. Multivariate analyses were performed in R (v4.3.2) using ropls (v1.34.0). PCA and PLS-DA were used to evaluate overall metabolic profile separation among the three groups, and the PLS-DA model was evaluated by 200-permutation testing. Pairwise OPLS-DA models were used to derive variable-importance-in-projection (VIP) scores for differential metabolite screening. For each pairwise comparison, two-sided *t*-tests were performed on log-transformed metabolite intensities. Metabolites with OPLS-DA VIP > 1.0 and nominal *p* < 0.05 were retained as differential features for exploratory analysis; Benjamini–Hochberg-adjusted *p*-values were additionally calculated and reported but were not used as the primary screening criterion. Pathway enrichment analysis was conducted in MetaboAnalyst 5.0 using the KEGG metabolic pathway library, and the potential dietary or microbial origin of differential metabolites was explored using MetOrigin.

### Renal transcriptomics

Total RNA was isolated from kidney tissue using TRIzol reagent (Invitrogen, USA) and treated with DNase I to remove genomic DNA contamination; RNA quality was assessed using a Bioanalyzer 2100. mRNA libraries were prepared using the VAHTS Universal V6 RNA-seq Library Prep Kit (Vazyme, China) and sequenced as 150-bp paired-end reads on an Illumina sequencing platform. Raw reads were quality-filtered using fastp (v0.19.5). Clean reads were aligned to the mouse reference genome GRCm39 (mm39) using STAR (v2.7.10b), and transcript assembly and gene-level count matrices were generated using StringTie (v1.3.5) and prepDE. Differential expression analysis was performed with DESeq2 (v1.10.1). For both model versus control and JPZS-H versus model, DEGs were defined as genes with nominal *p* < 0.05 and |log2(fold change)| > 1. The heat map was generated from the union of the two DEG lists. Expression values were log2-transformed as log2(expression + 1) and standardized to row-wise *z* scores; genes were clustered by average linkage using correlation distance, and samples were displayed by experimental group. Gene Ontology and KEGG overrepresentation analyses were performed using clusterProfiler. Directional KEGG analyses used genes upregulated in model versus control and genes downregulated in JPZS-H versus model. For each analysis, the 10 pathways with the smallest nominal enrichment *p*-values were selected for visualization; Benjamini–Hochberg-adjusted *p*-values were used to assess significance after multiple-testing correction. Gene set enrichment analysis (GSEA) was performed against KEGG gene sets using 1,000 permutations. Pathways with |normalized enrichment score (NES)| > 1.5 and FDR *q* < 0.25 were treated as exploratory enrichment signals and prioritized for targeted follow-up.

### Quantification of renal glycolytic intermediates and target validation

Lactate and pyruvate concentrations in kidney homogenate supernatants were measured using colorimetric kits (Nanjing Jiancheng Bioengineering Institute, A019-2–1 and A081-1-1, respectively) at 530 nm and 505 nm according to the manufacturer’s instructions; concentrations were normalized to protein content, and the lactate-to-pyruvate ratio was calculated from matched kidney samples after expressing both concentrations in equivalent molar units per unit protein. For qPCR, total RNA was reverse-transcribed using the PrimeScript RT Reagent Kit with gDNA Eraser (TaKaRa (Dalian, China), RR047A), and amplification was performed on a PikoReal 96 system (Thermo Scientific, Vantaa, Finland) with Novostart SYBR qPCR SuperMix Plus (Novoprotein (Suzhou, China), E096-01B); reactions were performed in a 10-μL volume with an initial denaturation at 95°C for 1 min, followed by 40 cycles of 95°C for 20 s and 60°C for 1 min, followed by melting-curve analysis. Renal Hk3 and Ldhal6b expression was quantified in six independent mice per group. Primer sequences are provided in **Supplementary Table S2**, and β-actin served as the reference gene for the 2^–ΔΔCt^ calculation. For western blotting, kidney lysates from control, model, and JPZS-H mice (*n* = 3 independent animals per group) were resolved by 10% SDS–PAGE, transferred to PVDF membranes, and probed with antibodies against HK3 (1:5,000; Proteintech, 67803-1-Ig; approximately 99 kDa), LDHAL6B (1:2,000; Thermo Fisher Scientific, PA5-69998), and GAPDH (1:2,000; Zsbio (Beijing, China), TA-08), followed by HRP-conjugated secondary antibodies (1:20,000; Zsbio, ZB-2305/ZB-2301); band intensities were quantified with ImageJ and normalized to GAPDH.

### Multi-omics integration

Integrative analysis of the gut microbiota, plasma metabolome, and renal transcriptome was performed using matched data from 18 mice (control, model, and JPZS-H groups; *n* = 6 per group). Fecal, EDTA-anticoagulated plasma, and kidney samples were derived from the same individual animals and matched by animal identity across the three omics layers. Features were first preselected by retaining those showing disease-associated alterations and treatment-responsive patterns: differential metabolites were defined by VIP > 1.0 in OPLS-DA and nominal *p* < 0.05; differential bacterial genera were identified by LEfSe (*p* < 0.05, LDA score > 2.0); and kidney genes were restricted to glycolysis/gluconeogenesis-related candidates included in the integrative analysis. Pairwise Spearman correlation coefficients were calculated across the 18 matched animals between the relative abundances of selected genera, the levels of JPZS-responsive metabolites, and the expression of glycolysis-related renal genes. Correlation heat maps were generated to visualize pairwise cross-omics associations, with nominal *p* < 0.05 used to indicate nominal statistical significance. For the three-layer network, associations with |*ρ*| > 0.60 and nominal *p* < 0.05 were retained, and key nodes from the retained associations were selected for network visualization. The cross-omics analysis was used for exploratory integration of the three data layers.

### Statistics

Data are expressed as mean ± SEM unless otherwise stated. Normality and homogeneity of variances were assessed by the Shapiro–Wilk test and Levene’s test, respectively. Normally distributed data with equal variances were analyzed by one-way ANOVA followed by Tukey’s *post hoc* test for all pairwise comparisons; non-normally distributed data were analyzed by the Kruskal–Wallis test with Dunn’s multiple comparison. Multiplicity control and statistical thresholds for omics analyses were analysis-specific and are described in the corresponding subsections. Differential-metabolite screening was based on a nominal two-sided *p* < 0.05 together with OPLS-DA VIP > 1.0, while Benjamini–Hochberg-adjusted *p*-values were additionally calculated and reported but were not used as the primary screening criterion. RNA-seq DEG selection and GSEA were evaluated using the thresholds specified above, whereas cross-omics correlation analyses were based on Spearman correlation coefficients and nominal *p*-values and were interpreted as exploratory. Statistical analyses were performed in SPSS 26.0 and the analysis-specific R packages described above; figures were generated with GraphPad Prism 8 and relevant R packages. No formal *a priori* power calculation was performed. The sample size of six animals per group was selected based on previous studies using the MRL/lpr model and experimental feasibility. Accordingly, multi-omics integration and correlation analyses were considered exploratory and interpreted cautiously. A two-sided *p* < 0.05 was considered statistically significant unless otherwise specified.

## Results

### JPZS attenuates systemic autoimmunity and lupus-associated renal injury

Compared with C57BL/6 controls, untreated MRL/lpr mice showed the expected autoimmune and renal phenotype, with marked elevations in serum ANA and anti-dsDNA antibodies, higher serum creatinine and urine protein-to-creatinine ratio (UPCR), and an increased kidney index (**Figures 1A–E**). All three JPZS dose groups exhibited significantly lower ANA and anti-dsDNA concentrations than the lupus model group, with reductions most pronounced in the high-dose group (**Figures 1A, B**). Serum creatinine and UPCR were similarly decreased, and the kidney index was reduced in JPZS-treated mice (**Figures 1C–E**), consistent with improvement in renal injury-related indices. Prednisone acetate (PED), included as a positive control, also significantly reduced autoantibody levels and renal functional indices (**Supplementary Figure S2**).

**Figure 1:**
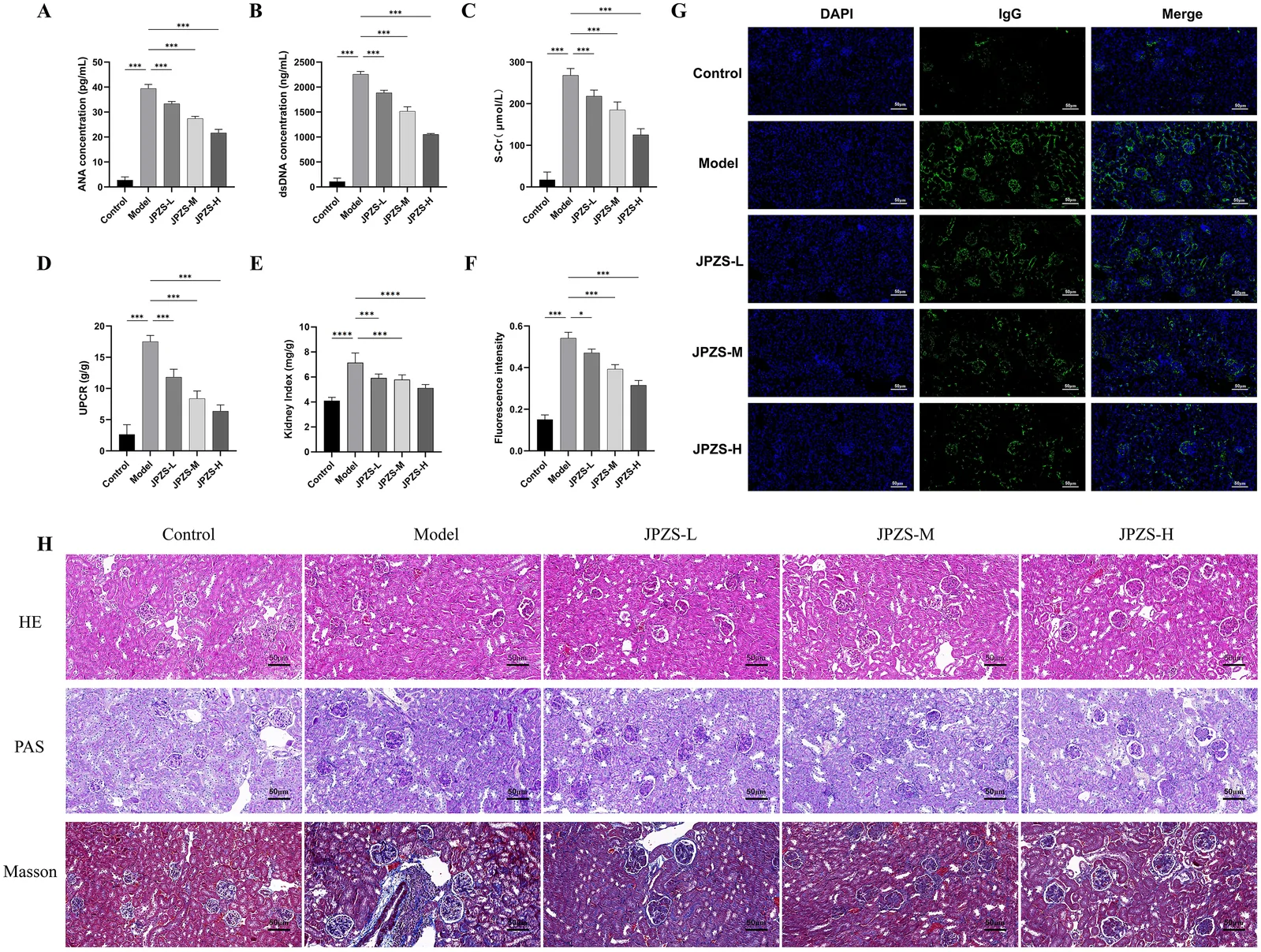
JPZS attenuates systemic autoimmunity and renal injury in MRL/lpr mice. **(A–E)** Serum ANA, anti-dsDNA antibody, S-Cr, UPCR, and kidney index in the control, model, JPZS-L, JPZS-M, and JPZS-H groups. Data are mean ± SEM (*n* = 6 independent mice per group). **(F)** Glomerular IgG fluorescence intensity (*n* = 3 independent mice per group). For quantitative comparisons in **(A–F)**, one-way ANOVA with Tukey’s *post hoc* test was used; ^*^p < 0.05, ^***^p < 0.001, ^****^p < 0.0001, as indicated. **(G)** Representative renal IgG immunofluorescence images (green signal; nuclei counterstained with DAPI in blue). **(H)** Representative kidney sections stained with H&E, PAS, and Masson’s trichrome. Scale bars: 50 µm.

Immunofluorescence staining revealed intense glomerular IgG deposition in lupus model kidneys, which was significantly reduced in all three JPZS dose groups, with the largest reduction observed in the high-dose group (**Figures 1F, G**). Representative histopathological sections showed mesangial hypercellularity, capillary loop narrowing, and inflammatory cell infiltration on H&E and PAS staining in model kidneys, together with increased collagen accumulation on Masson’s trichrome staining; these histopathological abnormalities appeared attenuated following JPZS treatment, with the most evident improvement in the high-dose group (**Figure 1H**). Collectively, these findings indicate that JPZS alleviates systemic autoimmunity and lupus-associated renal injury in MRL/lpr mice across the tested dose groups.

### Renal transcriptomic profiling identifies disease-associated immune activation and JPZS-associated transcriptional changes

Renal transcriptomic profiling identified substantial gene-expression alterations in MRL/lpr kidneys and a smaller set of changes associated with JPZS treatment (**Figures 2A, B**). Using the differential-expression threshold of nominal *p* < 0.05 and |log2 fold change| > 1, 770 genes were upregulated and 499 were downregulated in model mice compared with control mice. In the JPZS-H versus model comparison, 73 genes were upregulated and 50 were downregulated. A heat map constructed from the union of the two final pairwise DEG lists, comprising 1,348 unique genes, showed distinct expression patterns among the control, model, and JPZS-H groups (**Figure 2C**).

**Figure 2:**
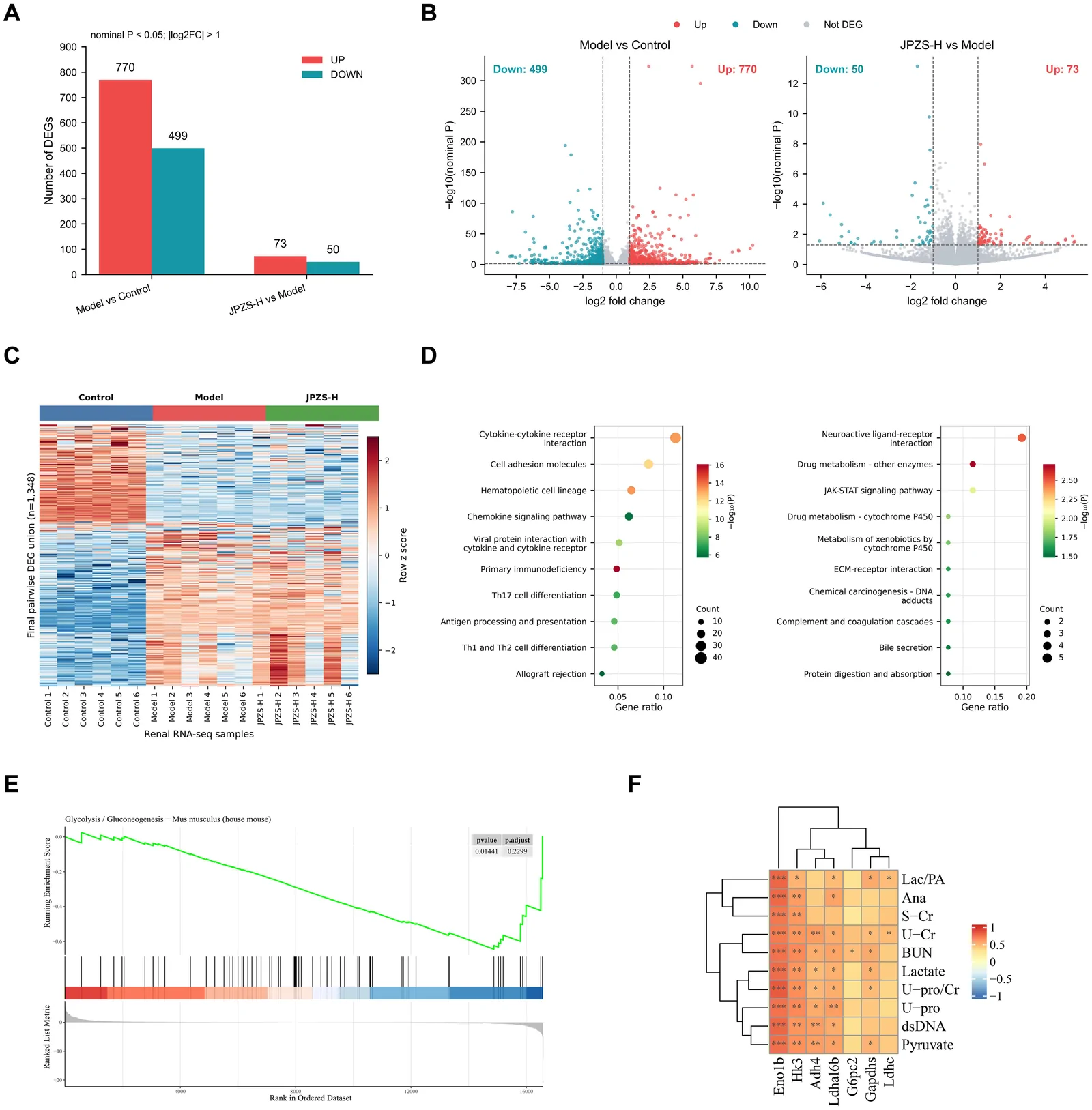
Renal transcriptomic changes associated with disease and JPZS treatment in MRL/lpr mice. **(A)** Numbers of up- and downregulated DEGs in model versus control and JPZS-H versus model (nominal *p* < 0.05 and |log2FC| > 1). **(B)** Volcano plots for the two comparisons; red, blue, and gray indicate upregulated, downregulated, and non-DEGs, respectively. **(C)** Row-standardized expression heat map of the union of the two final pairwise DEG lists (1,348 genes; *n* = 6 mice per group). **(D)** KEGG enrichment analysis of genes upregulated in model versus control (left) and downregulated in JPZS-H versus model (right). Dot size represents gene count, and color represents −log10(nominal *p*). **(E)** GSEA plot of the KEGG glycolysis/gluconeogenesis pathway in JPZS-H versus model (NES = −1.563, nominal *p* = 0.014, FDR = 0.230). **(F)** Spearman correlation heat map relating renal glycolysis-related genes to renal functional indices, serological markers, and renal lactate/pyruvate-related measurements across 18 matched mice; ^*^p < 0.05 and ^**^p < 0.01.

KEGG overrepresentation analysis of the genes upregulated in model versus control kidneys identified prominent immune-related pathways, including cytokine–cytokine receptor interaction, cell adhesion molecules, hematopoietic cell lineage, chemokine signaling, primary immunodeficiency, Th17 cell differentiation, antigen processing and presentation, and Th1 and Th2 cell differentiation (**Figure 2D**, left). These pathways remained significant after multiple-testing correction. Analysis of the 50 genes downregulated in JPZS-H versus model kidneys identified nominally enriched terms related to neuroactive ligand–receptor interaction, drug and xenobiotic metabolism, JAK–STAT signaling, extracellular matrix–receptor interaction, complement and coagulation cascades, and bile secretion (**Figure 2D**, right). However, none of the treatment-associated KEGG terms remained significant after multiple-testing correction; therefore, these results were interpreted as exploratory.

GSEA provided additional evidence of directionally opposing pathway-level changes between disease development and JPZS treatment (**Table 1**). The systemic lupus erythematosus pathway was positively enriched in model versus control kidneys (NES = 1.950, nominal *p* < 0.001, FDR < 0.001) and negatively enriched in JPZS-H versus model kidneys (NES = −1.784, nominal *p* < 0.001, FDR = 0.045). Glycolysis/gluconeogenesis showed a similar directionally opposing pattern, with positive enrichment in model versus control (NES = 1.556, nominal *p* = 0.045, FDR = 0.174) and negative enrichment in JPZS-H versus model (NES = −1.563, nominal *p* = 0.014, FDR = 0.230; **Figure 2E**). Both glycolysis/gluconeogenesis comparisons met the prespecified exploratory GSEA criterion of FDR < 0.25 but did not meet the conventional FDR < 0.05 threshold. Accordingly, the glycolysis-related signal was treated as hypothesis-generating and was examined further using targeted measurements.

**Table 1:** Representative renal GSEA pathways showing opposite enrichment patterns between disease and treatment comparisons.

KEGG ID	Pathway	Set size	JPZS-H vs. model			Model vs. control		
			NES	Nominal *p*	FDR	NES	Nominal *p*	FDR
mmu00010	Glycolysis/gluconeogenesis	63	−1.563	0.014	0.230	1.556	0.045	0.174
mmu04514	Cell adhesion molecules	157	−1.543	0.003	0.13	1.724	<0.001	<0.001
mmu04612	Antigen processing and presentation	70	−1.54	0.013	0.229	1.664	0.001	0.01
mmu04713	Circadian entrainment	92	1.55	0.004	0.13	−1.517	0.024	0.129
mmu04727	GABAergic synapse	79	1.606	0.003	0.13	−1.614	0.013	0.079
mmu05032	Morphine addiction	83	1.694	<0.001	0.069	−1.565	0.019	0.11
mmu05140	Leishmaniasis	69	−1.629	0.003	0.13	1.707	<0.001	0.007
mmu05144	Malaria	58	−1.511	0.016	0.229	1.74	<0.001	0.004
mmu05168	Herpes simplex virus 1 infection	177	−1.578	0.001	0.106	1.74	<0.001	<0.001
mmu05320	Autoimmune thyroid disease	45	−1.548	0.011	0.229	1.579	0.006	0.041
mmu05322	Systemic lupus erythematosus	55	−1.784	<0.001	0.045	1.95	<0.001	<0.001

The NES sign is defined according to the comparison indicated in the column heading: a positive NES denotes enrichment toward the first-named group, whereas a negative NES denotes enrichment toward the second-named group. The displayed pathways showed opposite NES directions between the two comparisons and met the exploratory GSEA criterion of |NES| > 1.5 and FDR < 0.25 in both comparisons. Findings with FDR ≥ 0.05 were interpreted as exploratory. NES, normalized enrichment score; FDR, false discovery rate.

Spearman correlation analysis further showed that the expression levels of selected glycolysis-related genes were associated with renal functional indices, serological markers, and renal lactate- and pyruvate-related measurements (**Figure 2F**). These associations supported the selection of glycolysis-related candidates for downstream validation but were interpreted as correlative rather than causal.

### JPZS reshapes gut microbial community structure without significantly restoring alpha diversity

Fecal 16S rRNA sequencing revealed a profound loss of gut microbial diversity in MRL/lpr mice. The Chao1 richness index and Shannon diversity index were both markedly lower in the model group than in healthy controls (**Figures 3A, B**), indicating lower estimated richness and Shannon diversity. JPZS treatment did not significantly increase either Chao1 richness (model vs. JPZS-H, *p* = 1.000) or Shannon diversity (*p* = 0.132), indicating that the alpha diversity deficit persisted after treatment.

**Figure 3:**
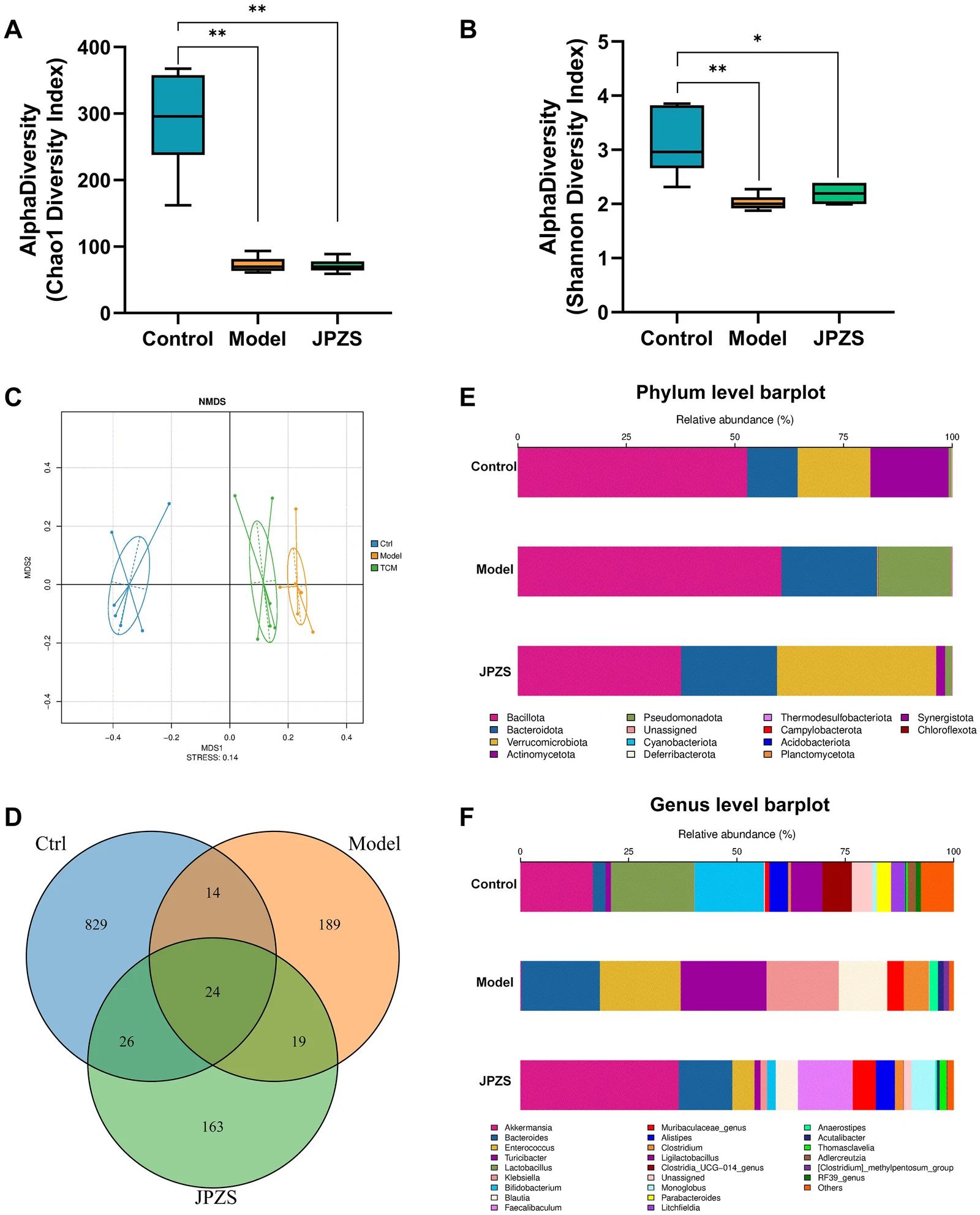
JPZS reshapes gut microbial community structure in MRL/lpr mice. **(A, B)** Alpha diversity assessed by the Chao1 richness index **(A)** and Shannon diversity index **(B)** in the control, model, and JPZS-H groups (*n* = 6 independent mice per group). Group differences were analyzed using the Kruskal–Wallis test followed by Dunn’s multiple-comparisons test; significance is indicated by the brackets shown in the image. **(C)** NMDS ordination based on Bray–Curtis dissimilarity showing separation of overall microbial community structure across groups (STRESS = 0.14; PERMANOVA *R*² = 0.771, *p* < 0.001). **(D)** Venn diagram showing shared and unique ASVs among the three groups. **(E, F)** Stacked bar charts of relative abundance at the phylum **(E)** and genus **(F)** levels.

Beta diversity analysis based on Bray–Curtis dissimilarity showed clear separation of all three groups by NMDS ordination (STRESS = 0.14); PERMANOVA confirmed that group membership explained a substantial proportion of the variance in community composition (*R*² = 0.771, *F* = 25.199, *p* < 0.001, 9,999 permutations), and ANOSIM similarly indicated that intergroup distances significantly exceeded intragroup distances (*R* = 1.000, *p* < 0.001) (**Figure 3C**). Venn analysis of ASVs further illustrated the compositional shifts: each group harbored a substantial number of unique ASVs, while JPZS-treated mice shared more ASVs with controls than did untreated lupus mice, indicating a JPZS-associated shift in community composition (**Figure 3D**).

At the phylum level, control mice were dominated by Bacillota and Bacteroidota, whereas lupus model mice showed expansion of Pseudomonadota and contraction of Verrucomicrobiota (**Figure 3E**). JPZS treatment reduced Pseudomonadota abundance and markedly increased Verrucomicrobiota, with its relative abundance exceeding that observed in controls. At the genus level, lupus model mice exhibited increased relative abundances of *Klebsiella*, *Enterococcus*, and *Clostridioides* alongside depletion of *Akkermansia* and several anaerobic commensals, while JPZS-treated mice showed a distinct compositional profile characterized by increased *Akkermansia* and lower relative abundances of several model-enriched genera (**Figure 3F**). Collectively, these data indicate that JPZS substantially remodeled gut microbial community structure and taxonomic composition despite the absence of significant recovery in Chao1 or Shannon alpha diversity, setting the stage for taxon-specific analyses in the following section.

### JPZS selectively enriches *Akkermansia* and reshapes gut microbial composition and predicted carbohydrate metabolism

LEfSe analysis identified taxon-level signatures discriminating the three groups. The cladogram revealed distinct bacterial clades associated with each condition: control mice were characterized by several Lachnospiraceae-related lineages, lupus model mice by expansions within Enterobacteriaceae and clostridial groups, and JPZS-treated mice by a clade spanning phylum Verrucomicrobiota to genus *Akkermansia* (**Figure 4A**). The LDA bar plot confirmed the highest discriminatory scores for *Akkermansia* and its parent taxa in the JPZS group, alongside *Faecalibaculum*, whereas *Enterococcus*, *Klebsiella*, and a *Clostridium*-related taxon (*Clostridium* methylpentosum group) were among the top discriminators in the model group (**Figure 4B**).

**Figure 4:**
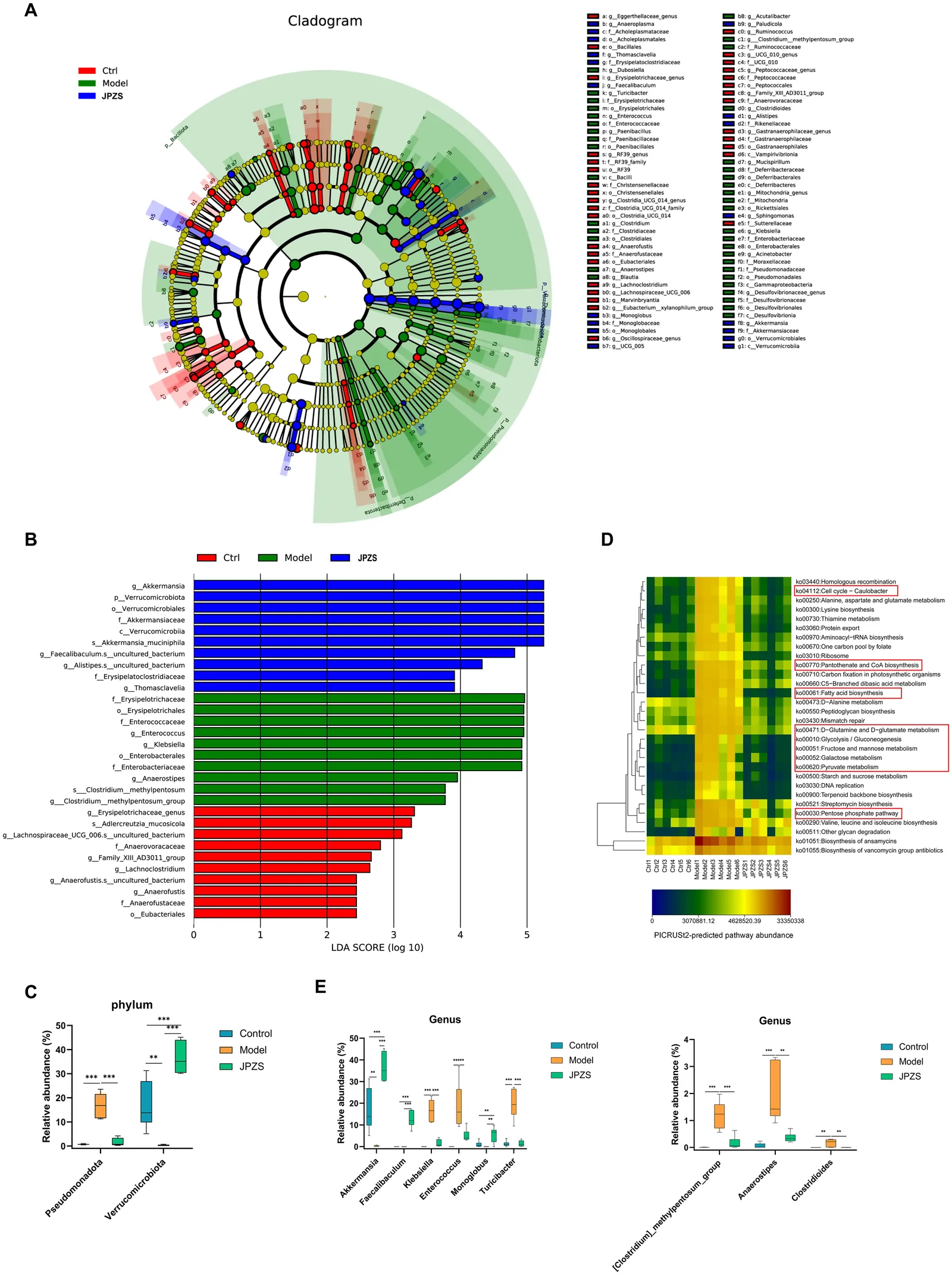
JPZS selectively enriches *Akkermansia*, reshapes gut microbial composition, and is associated with changes in predicted carbohydrate-metabolism pathways in MRL/lpr mice. **(A)** LEfSe cladogram showing differentially enriched bacterial clades among the control, model, and JPZS-H groups (LDA score > 2.0, *p* < 0.05). **(B)** LDA score bar plot of discriminative taxa identified by LEfSe. **(C)** Relative abundance of representative phyla (Pseudomonadota and Verrucomicrobiota) across groups; *n* = 6 independent mice per group; ^**^p < 0.01, ^***^p < 0.001; one-way ANOVA with Tukey’s *post hoc* test. **(D)** Heat map of PICRUSt2-predicted KEGG functional pathway abundances, highlighting JPZS-associated changes in carbohydrate and glycolysis-related metabolic programs (exploratory 16S-based functional prediction; color scale indicates predicted pathway abundance). **(E)** Relative abundance of representative genera across groups; *n* = 6 independent mice per group; ^**^p < 0.01, ^***^p < 0.001.

Quantitative comparisons corroborated these findings across taxonomic levels. At the phylum level, Pseudomonadota was significantly expanded in lupus model mice and reduced by JPZS treatment, whereas Verrucomicrobiota was nearly absent in the model group, present at intermediate levels in controls, and markedly enriched in JPZS-treated mice to levels significantly exceeding both other groups (**Figure 4C**). At the genus level, *Akkermansia* mirrored this pattern—depleted in lupus mice and markedly enriched following JPZS treatment, reaching levels above those observed in controls—while JPZS also increased *Faecalibaculum* and *Monoglobus* and suppressed the lupus-associated overgrowth of *Klebsiella*, *Enterococcus*, *Turicibacter, Anaerostipes*, [*Clostridium*] methylpentosum group, and *Clostridioides* (**Figure 4E**). Together, these data indicate that JPZS treatment was associated with higher relative abundance of *Akkermansia* and changes in multiple model-associated genera.

PICRUSt2-based functional prediction indicated that the microbiota of lupus model mice had higher predicted contributions to glycolysis/gluconeogenesis, pyruvate metabolism, the pentose phosphate pathway, and galactose metabolism relative to controls, and that JPZS treatment was associated with lower predicted abundances of these carbohydrate-metabolism pathways relative to the model group (**Figure 4D**). Given the marker–gene basis of these predictions, this analysis is treated as exploratory; these predicted functional differences should not be interpreted as direct measurements of microbial metabolic activity.

### JPZS remodels the plasma metabolome with emphasis on amino-acid and central carbon metabolism

Untargeted plasma metabolomics revealed distinct metabolic profiles across the three groups. In the PLS-DA score plot, control, lupus model, and JPZS-treated mice formed three clearly separated clusters (p1 = 17.6%, p2 = 9.01%; **Figure 5A**), with the three-group PLS-DA model yielding *R*²*Y*(cum) = 0.994 and *Q*²(cum) = 0.833. In the 200-permutation testing, both p*R*²*Y* and p*Q*² were 0.005 (**Figure 5B**), supporting the stability of the multivariate model.

**Figure 5:**
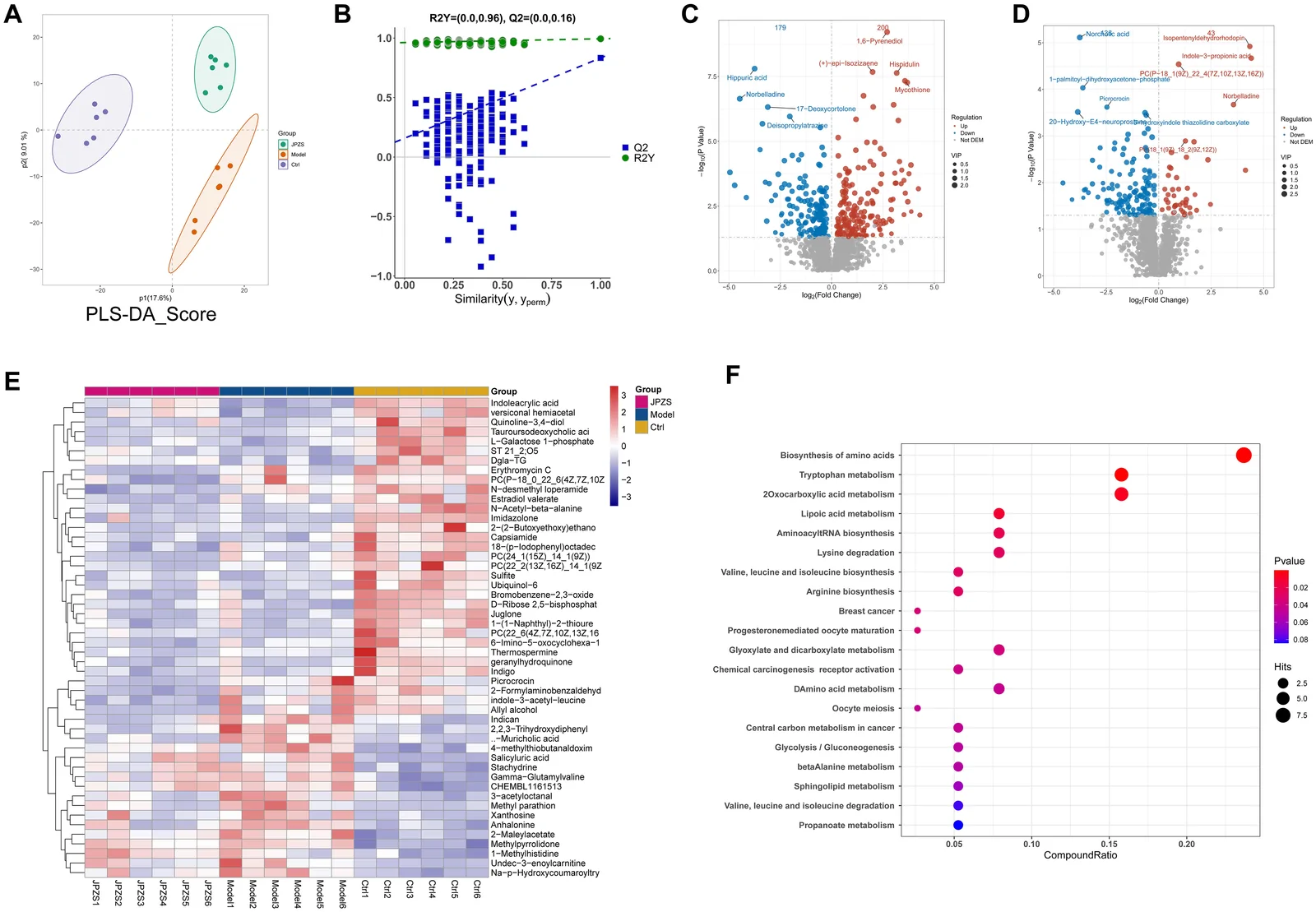
JPZS remodels the plasma metabolome in lupus-prone MRL/lpr mice. **(A)** PLS-DA score plot showing separation of EDTA-plasma metabolic profiles among the control, model, and JPZS-H groups (p1 = 17.6%, p2 = 9.01%, *n* = 6 independent mice per group). **(B)** PLS-DA permutation test (200 permutations); the regression intercepts were *R*²*Y* = 0.96 and *Q*² = 0.16, while the original three-group model yielded *R*²*Y*(cum) = 0.994 and *Q*²(cum) = 0.833 (p*R*²*Y* = p*Q*² = 0.005). **(C)** Volcano plot of differential metabolites for model versus control (200 upregulated and 179 downregulated features). **(D)** Volcano plot of differential metabolites for JPZS-H versus model (43 upregulated and 136 downregulated features). **(E)** Heat map of differential plasma metabolic features across all samples. **(F)** KEGG pathway enrichment analysis of JPZS-responsive differential metabolites. Differential metabolites were defined by pairwise OPLS-DA VIP > 1.0 and nominal two-sided *p* < 0.05; Benjamini–Hochberg-adjusted *p*-values were additionally calculated and reported but were not used as the primary feature-selection criterion.

Compared with controls, lupus model mice displayed widespread plasma metabolic alterations, with 200 upregulated and 179 downregulated differential features based on OPLS-DA VIP > 1.0 and nominal *p* < 0.05 (**Figure 5C**). JPZS treatment substantially reshaped this pattern: the JPZS-H versus model contrast identified 43 upregulated and 136 downregulated differential features under the same screening criterion (**Figure 5D**). A heat map of the differential features showed distinct group-associated metabolic patterns across the control, model, and JPZS-H samples (**Figure 5E**).

Pathway enrichment analysis of JPZS-responsive metabolites highlighted alterations in amino-acid and central carbon metabolic pathways (**Figure 5F**). Biosynthesis of amino acids (FDR = 0.00286) and tryptophan metabolism (FDR = 0.0458) remained significant after Benjamini–Hochberg correction. Additional pathways, including 2-oxocarboxylic acid metabolism, glyoxylate and dicarboxylate metabolism, and glycolysis/gluconeogenesis, reached nominal *p* < 0.05 but did not meet FDR < 0.05 and were therefore interpreted as exploratory. Together, these findings indicate that JPZS treatment was associated with broad remodeling of the circulating plasma metabolome, particularly involving amino-acid metabolism and selected central carbon metabolic features.

### Multi-omics integration identifies exploratory associations among gut genera, plasma metabolites, and renal markers

Cross-omics correlation heat maps first linked JPZS-responsive taxa with plasma metabolites (**Figure 6A**). *Akkermansia*, *Faecalibaculum*, and *Anaerostipes* formed a cluster that associated positively with amino-acid derivatives, bile acids, and carnitine species, whereas *Klebsiella*, *Turicibacter*, *Enterococcus*, and a *Clostridium*-related taxon (*Clostridium* methylpentosum group) aligned with metabolites selectively elevated in the model group, including p-cresol, neuroprostane, and several indole derivatives. A second heat map relating these metabolites to renal glycolytic genes showed that HK3, LDHAL6B, and aldolase C (ALDOC) tracked the same metabolite module characterizing the lupus phenotype (**Figure 6A**), indicating coordinated cross-omics associations rather than directional or causal relationships.

**Figure 6:**
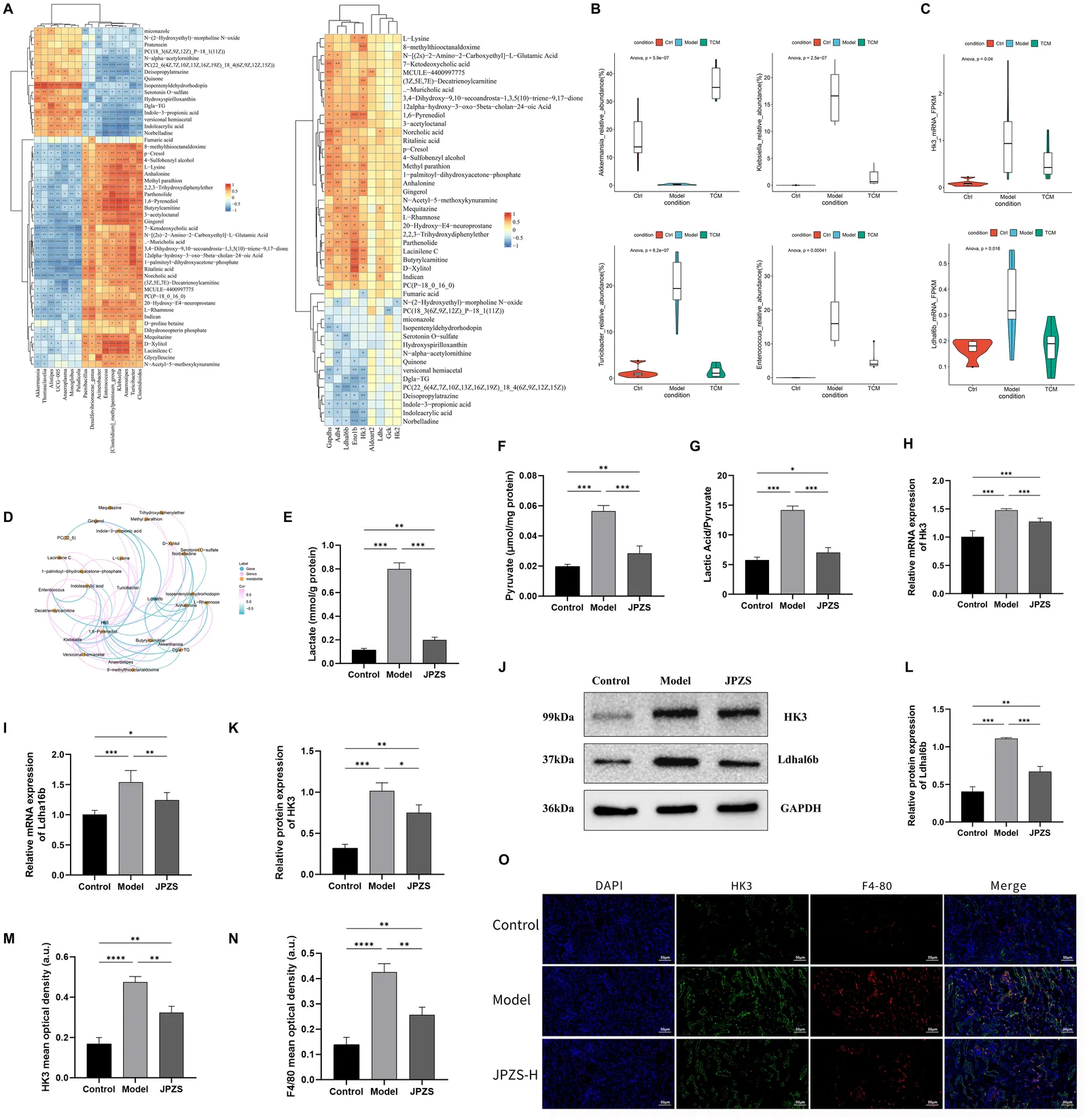
Multi-omics integration identifies coordinated *Akkermansia*-, plasma-metabolite-, and renal HK3-associated changes following JPZS treatment in MRL/lpr mice. **(A)** Spearman correlation heat maps showing pairwise associations between selected gut bacterial genera and plasma metabolites, and between plasma metabolites and renal glycolysis-related genes, across the 18 matched animals. Nominal *p* < 0.05 was used to denote nominal statistical significance. **(B)** Relative abundance of representative bacterial genera across the control, model, and JPZS-H groups (*n* = 6 independent mice per group). **(C)** Renal expression levels of *Hk3* and *Ldhal6b* across groups derived from RNA-seq data and expressed as FPKM (*n* = 6 independent mice per group). **(D)** Exploratory three-layer Spearman correlation network integrating selected bacterial genera, plasma metabolites, and renal genes. Edges represent associations with |*r*| > 0.60 and nominal *p* < 0.05. The network is intended for hypothesis generation and does not imply directionality or causality. **(E–G)** Targeted quantification of renal lactate **(E)**, pyruvate **(F)**, and lactate/pyruvate ratio **(G)** (*n* = 6 independent mice per group). **(H, I)** qPCR validation of renal *Hk3* **(H)** and *Ldhal6b* **(I)** mRNA expression normalized to β-actin (*n* = 6 independent mice per group). **(J)** Representative immunoblot of HK3 and LDHAL6B protein with GAPDH as loading control. **(K, L)** Densitometric quantification of HK3 **(K)** and LDHAL6B **(L)** protein levels normalized to GAPDH (*n* = 3 independent mice per group). **(M)** Quantification of renal HK3 mean optical density by immunofluorescence. **(N)** Quantification of renal F4/80 mean optical density by immunofluorescence. **(O)** Representative dual-immunofluorescence images of HK3 (green) and F4/80 (red) in kidney sections from control, model, and JPZS-H mice; nuclei were counterstained with DAPI (blue). Merged images show the spatial distribution of HK3 and F4/80 signals. Scale bars: 50 μm. For **(M–O)**, *n* = 3 independent mice per group. Data are presented as mean ± SEM. Targeted quantitative comparisons were analyzed by one-way ANOVA followed by Tukey’s multiple-comparisons test. *p < 0.05, **p < 0.01, ***p < 0.001, ****p < 0.0001.

At the community level, *Akkermansia* was nearly undetectable in lupus mice but markedly enriched after JPZS treatment, while pathobionts including *Klebsiella*, *Turicibacter*, and *Enterococcus* expanded in the model group and were markedly suppressed by JPZS (**Figure 6B**). Bulk-kidney RNA-seq showed that Hk3 expression was higher in model than control kidneys and remained elevated in JPZS-H mice, whereas Ldhal6b showed little difference between control and model but was higher in JPZS-H mice (**Figure 6C**). These RNA-seq patterns were therefore considered separately from the subsequent targeted qPCR and protein measurements. A three-layer Spearman correlation network integrating genera, metabolites, and genes placed *Akkermansia* and *Anaerostipes* on the opposite pole from the pathobiont cluster, with HK3 and LDHAL6B connected to multiple microbial and metabolic features (**Figure 6D**), supporting an exploratory cross-omics association network rather than a regulatory or causal axis.

Targeted renal measurements further characterized JPZS-associated metabolic changes. Compared with controls, lupus mice showed markedly elevated renal lactate and pyruvate concentrations and an increased lactate/pyruvate ratio (**Figures 6E–G**). JPZS significantly reduced all three parameters towards control values; prednisone acetate (PED), included as a positive control, produced a similar reduction in renal lactate burden (**Supplementary Figure S3**). These endpoint measurements reflect renal lactate/pyruvate status and do not directly measure glycolytic flux.

Consistent with these metabolite data, qPCR showed that renal Hk3 and Ldhal6b mRNA expression was higher in model mice than in control mice and was significantly reduced following JPZS-H treatment (**Figures 6H, I**). Western blot analysis showed a similar pattern at the protein level, with increased HK3 and LDHAL6B abundance in model kidneys and lower levels following JPZS-H treatment (**Figures 6J–L**). Dual immunofluorescence further showed stronger HK3 and F4/80 fluorescence in model kidneys than in control kidneys, with overlapping HK3 and F4/80 signals evident in merged images. Both HK3 and F4/80 mean optical densities were significantly lower following JPZS-H treatment (**Figures 6M–O**).

Together, these multi-omics findings identify coordinated JPZS-associated changes in gut bacterial composition, plasma metabolites, renal lactate/pyruvate status, and targeted HK3 expression. These data support a putative *Akkermansia*–HK3–lactate immunometabolic association, while its directionality and causal relationships remain to be established experimentally.

## Discussion

This multi-omics study shows that JPZS treatment is associated with renoprotection in MRL/lpr lupus-prone mice and with coordinated changes across the gut microbiota, circulating metabolome, and kidney. JPZS reduced autoantibody titers, ameliorated proteinuria and renal dysfunction, and improved glomerular and tubulointerstitial pathology. These improvements were accompanied by *Akkermansia* enrichment, altered plasma amino-acid metabolism, and reductions in renal HK3 expression measured by targeted assays and in intrarenal lactate accumulation. Dual immunofluorescence further revealed spatial overlap between renal HK3 and F4/80 signals, with lower fluorescence intensities of both markers following JPZS-H treatment. By profiling fecal, plasma, and renal samples from the same animals, this study places these treatment-associated changes within a shared biological context and identifies candidate connections between intestinal ecology and renal immunometabolism.

The reduction in renal lactate accumulation is relevant to the metabolic requirements of inflammation in LN. Previous work showed that immune-complex stimulation induces macrophage glycolysis through mTOR and HIF-1α, and that glycolysis inhibition reduces inflammatory responses in antibody-mediated nephritis (9). Lactate has also been implicated in PBX1 lactylation and mesangial-cell proliferation in LN, extending its potential relevance beyond infiltrating immune cells (26). In this context, the lower renal lactate concentration and lactate/pyruvate ratio following JPZS treatment indicate a change in the intrarenal metabolic environment accompanying reduced tissue injury. The spatial overlap of HK3 with F4/80 connects the renal HK3 findings to the macrophage-associated inflammatory microenvironment in LN. This observation is consistent with the myeloid enrichment of HK3 and its reported role in supporting cell survival through non-glycolytic mechanisms (27), suggesting that the relevance of HK3 to renal inflammation may extend beyond glucose phosphorylation. The concurrent changes in renal HK3, F4/80, and lactate following JPZS treatment raise the possibility that improvement in the local metabolic environment accompanies changes in macrophage-associated inflammation. HK3 thus emerges as a candidate for exploring the relationship between myeloid-cell biology and renal metabolic alterations during the response to JPZS.

The different analytical layers refine this interpretation. The glycolysis/gluconeogenesis GSEA signal provided an exploratory rationale for targeted measurements but did not meet FDR < 0.05. Bulk RNA-seq also did not reproduce the treatment-associated decreases in Hk3 and Ldhal6b detected by qPCR, leaving an unresolved discrepancy between the expression assays. The treatment-associated reduction in renal HK3 was supported by qPCR, immunoblotting, and tissue immunofluorescence, with the latter additionally providing spatial information relative to F4/80-positive regions. LDHAL6B is retained as an exploratory expression marker; its changes do not establish a role in renal lactate production, which requires investigation of canonical LDHA/LDHB activity and metabolic flux. Together, the targeted findings support altered renal HK3 expression and lactate/pyruvate status, while the functional relationship between them remains a specific question for intervention studies.

Our gut microbiota findings position JPZS within the broader concept of a dysregulated gut–kidney axis in lupus nephritis. JPZS increased the relative abundance of *Akkermansia* and reduced that of *Klebsiella* and *Enterococcus* without significantly restoring Chao1 or Shannon diversity. *Akkermansia* abundance exceeded that in healthy controls, indicating a treatment-associated community configuration rather than a return to the control microbiota. This distinction suggests that changes in selected taxa may be more informative for understanding the response to JPZS than a general claim of microbial restoration. Experimental administration of *A. muciniphila* has improved intestinal barrier integrity, inflammatory cytokine profiles, and renal outcomes in MRL/lpr mice (17), providing biological support for prioritizing this genus. Our genus-level measurements identify *Akkermansia* as a candidate for follow-up, without resolving the species or strains responsible. PICRUSt2 predictions further suggest differences in microbial carbohydrate-metabolism potential, which can guide direct functional profiling of the altered community.

Our plasma metabolomics data complement these microbial observations by identifying circulating metabolic changes associated with JPZS treatment. Amino-acid biosynthesis and tryptophan metabolism remained enriched after multiple-testing correction, providing stronger pathway-level support than the nominal changes in central carbon metabolism. Human metabolomic studies have likewise identified circulating amino-acid and lipid differences between SLE and LN (28), although the specific profiles need not be identical across species or sample matrices. Experimental evidence linking altered microbial tryptophan catabolism to lupus autoimmunity makes this pathway particularly relevant to the gut–kidney connection (29). The cross-omics associations in our study prioritize metabolites for testing as potential intermediates between microbial composition and renal responses. Their origins and roles remain open: altered plasma abundance could reflect microbial metabolism, host metabolism, or changes in renal handling. Targeted metabolite quantification and tracing would help distinguish these possibilities.

These findings extend previous work on JPZS by placing its immunological effects alongside changes in intestinal ecology and circulating metabolism. Earlier studies linked JPZS to Treg/Th17 regulation through DNMT1-associated Foxp3 methylation and to macrophage polarization involving AMPK/ULK1 signaling (22, 23). The present study adds a set of microbial, metabolic, and renal readouts that can be examined together in subsequent intervention studies. The chemical profile of the preparation provides a starting point for fractionation, but does not identify which constituents account for *Akkermansia* enrichment or the renal changes. Prednisone also reduced renal lactate in this study, suggesting that this endpoint may accompany a broader response to treatment; its exclusion from multi-omics profiling prevents comparison of the microbial and metabolic responses to the two interventions. Clinically, the next step is to evaluate these candidate readouts in patients receiving JPZS as an adjunct to standard care. Prospective studies could test whether baseline microbiota composition or circulating metabolites are associated with treatment response before using these features to guide patient selection.

Several limitations should be acknowledged. First, experiments were performed in a small cohort of female MRL/lpr mice at a single disease stage, precluding assessment of sex-specific or temporal dynamics of immune–metabolic and microbial rewiring. The use of C57BL/6 controls also means that disease-versus-control differences include genetic background effects; treatment comparisons within MRL/lpr mice therefore provide the more direct evidence for JPZS-associated changes. The modest sample size and pooling of the three groups for correlation analysis make the networks sensitive to group separation and limit their generalizability. No formal *a priori* power calculation was performed, and the cross-omics correlations were evaluated using nominal *p*-values; accordingly, the resulting network should be regarded as hypothesis-generating and requires independent replication. Although dual immunofluorescence demonstrated spatial overlap between HK3 and F4/80, the separate quantification of their fluorescence intensities does not distinguish changes in macrophage abundance from changes in HK3 expression within macrophages. The cellular sources of the altered renal lactate pool also remain unresolved. Studies combining defined microbial colonization or microbiota transfer with cell-specific HK3 perturbation would test whether intestinal changes contribute to the renal response and whether HK3 is functionally involved. Within these boundaries, the study demonstrates renal improvement accompanied by microbial and metabolic remodeling and nominates the *Akkermansia*–HK3–lactate relationship for focused investigation in LN.

## Conclusion

JPZS ameliorated renal injury in MRL/lpr mice, accompanied by *Akkermansia* enrichment, remodeling of the plasma metabolome, and reduced renal HK3 expression in targeted assays and intrarenal lactate accumulation. Integrated profiling of matched samples identified associations among gut microbial composition, circulating metabolites, and renal gene expression, providing a framework for investigating gut–kidney immunometabolic interactions in lupus nephritis. These findings support further evaluation of JPZS and prioritize the putative *Akkermansia*–HK3–lactate relationship for functional investigation.

## Data Availability

The renal RNA-seq data have been deposited in the NCBI Gene Expression Omnibus (GEO) under accession number GSE314172 and will be released publicly upon publication. The 16S rRNA gene sequencing data have been deposited in the NCBI Sequence Read Archive (SRA) under BioProject PRJNA1381362 and will be released publicly upon publication. The plasma untargeted metabolomics data have been submitted to the Metabolomics Workbench (DataTrack ID 6900) and will be made publicly available upon publication.
