# Supplementary material for: Multi-omics profiling identifies a putative Akkermansia–HK3–lactate immunometabolic link associated with JPZS treatment in lupus nephritis
Source: Front Immunol. 2026 Oct 22;17:1873518. doi: 10.3389/fimmu.2026.1873518 (PMC13735744; doi:10.3389/fimmu.2026.1873518)
Supplement: Supplementary file 1 [file DataSheet1.pdf]

A

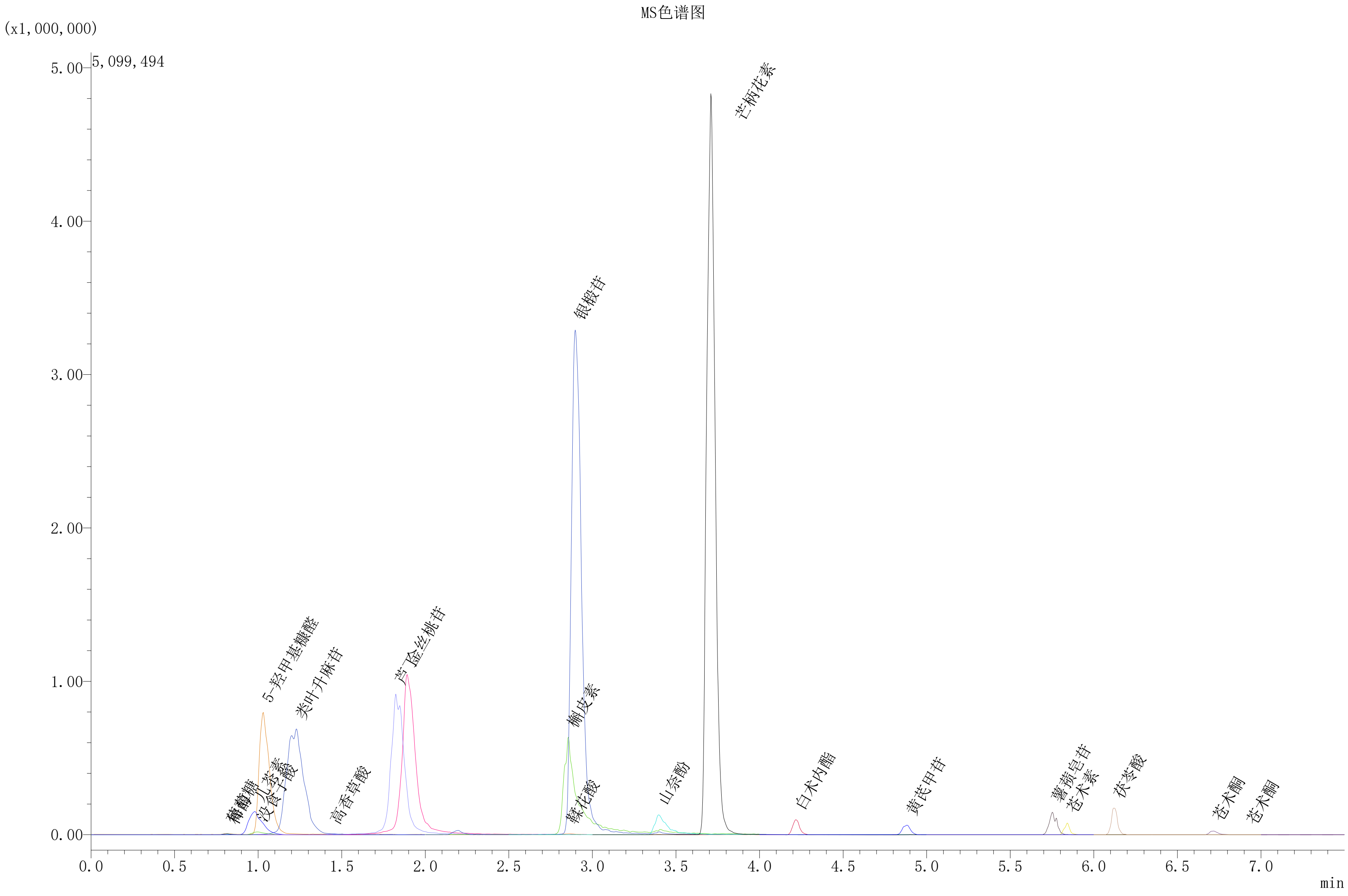

MS色谱图
(x1,000,000)
5,099,494
5.00
4.00
3.00
2.00
1.00
0.00
0.0
0.5
1.0
1.5
2.0
2.5
3.0
3.5
4.0
4.5
5.0
5.5
6.0
6.5
7.0
min
葡萄糖
儿茶素
没食子酸
5-羟甲基糠醛
类叶升麻苷
高香草酸
芦丁
金丝桃苷
银椴苷
槲皮素
鞣花酸
山奈酚
芒柄花素
白术内酯
黄芪甲苷
薯蓣皂苷
苍术素
茯苓酸
苍术酮
苍术酮

B

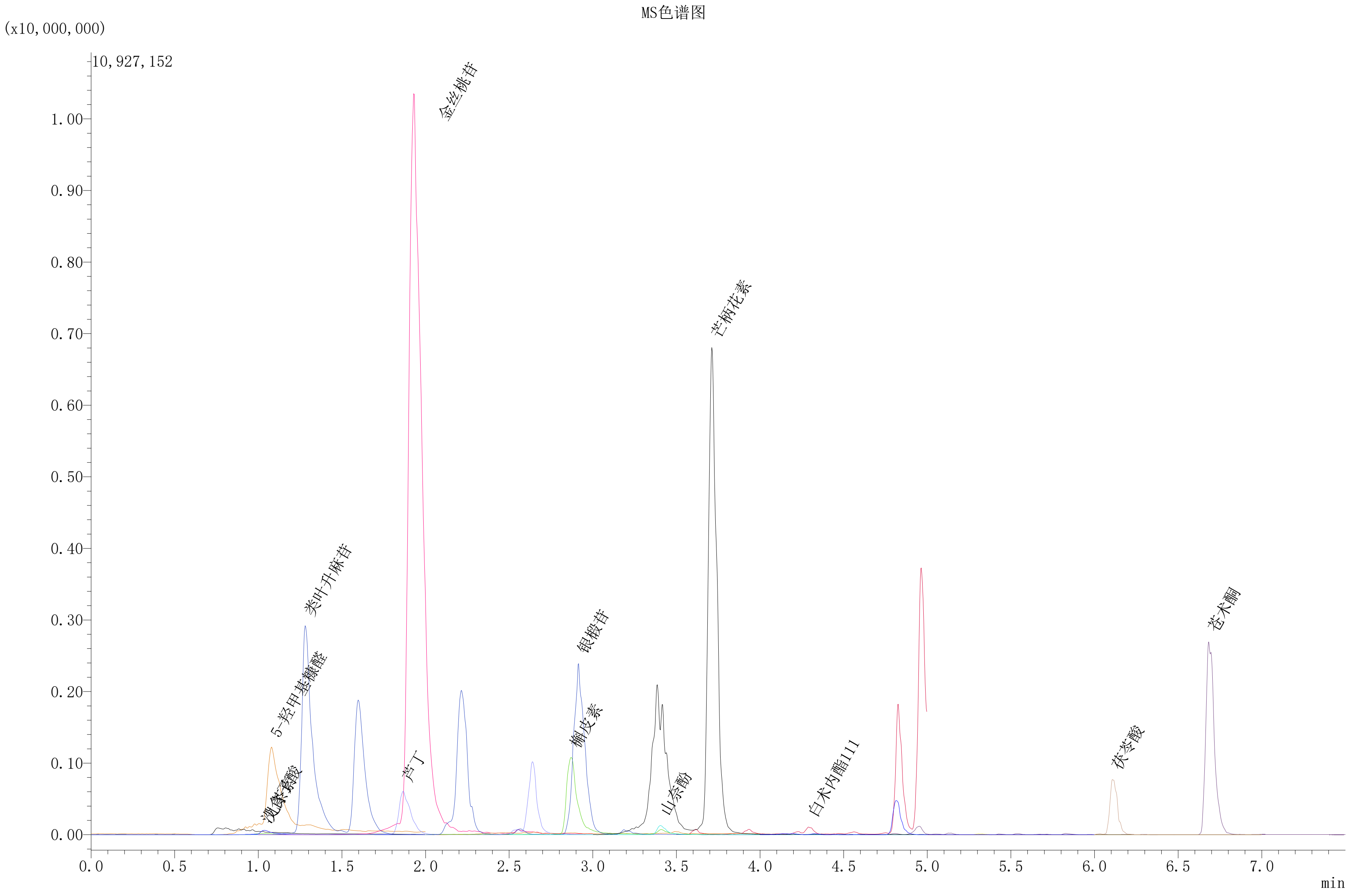

MS色谱图
(x10,000,000)
10,927,152
1.00
0.90
0.80
0.70
0.60
0.50
0.40
0.30
0.20
0.10
0.00
0.0
0.5
1.0
1.5
2.0
2.5
3.0
3.5
4.0
4.5
5.0
5.5
6.0
6.5
7.0
min
5-羟甲基糠醛
类叶升麻苷
芦丁
金丝桃苷
槲皮素
银椴苷
山奈酚
芒柄花素
白术内酯III
茯苓酸
苍术酮
