# Supplementary material for: Multi-omics profiling identifies a putative Akkermansia–HK3–lactate immunometabolic link associated with JPZS treatment in lupus nephritis
Source: Front Immunol. 2026 Oct 22;17:1873518. doi: 10.3389/fimmu.2026.1873518 (PMC13735744; doi:10.3389/fimmu.2026.1873518)
Supplement: Supplementary file 2 [file DataSheet2.pdf]

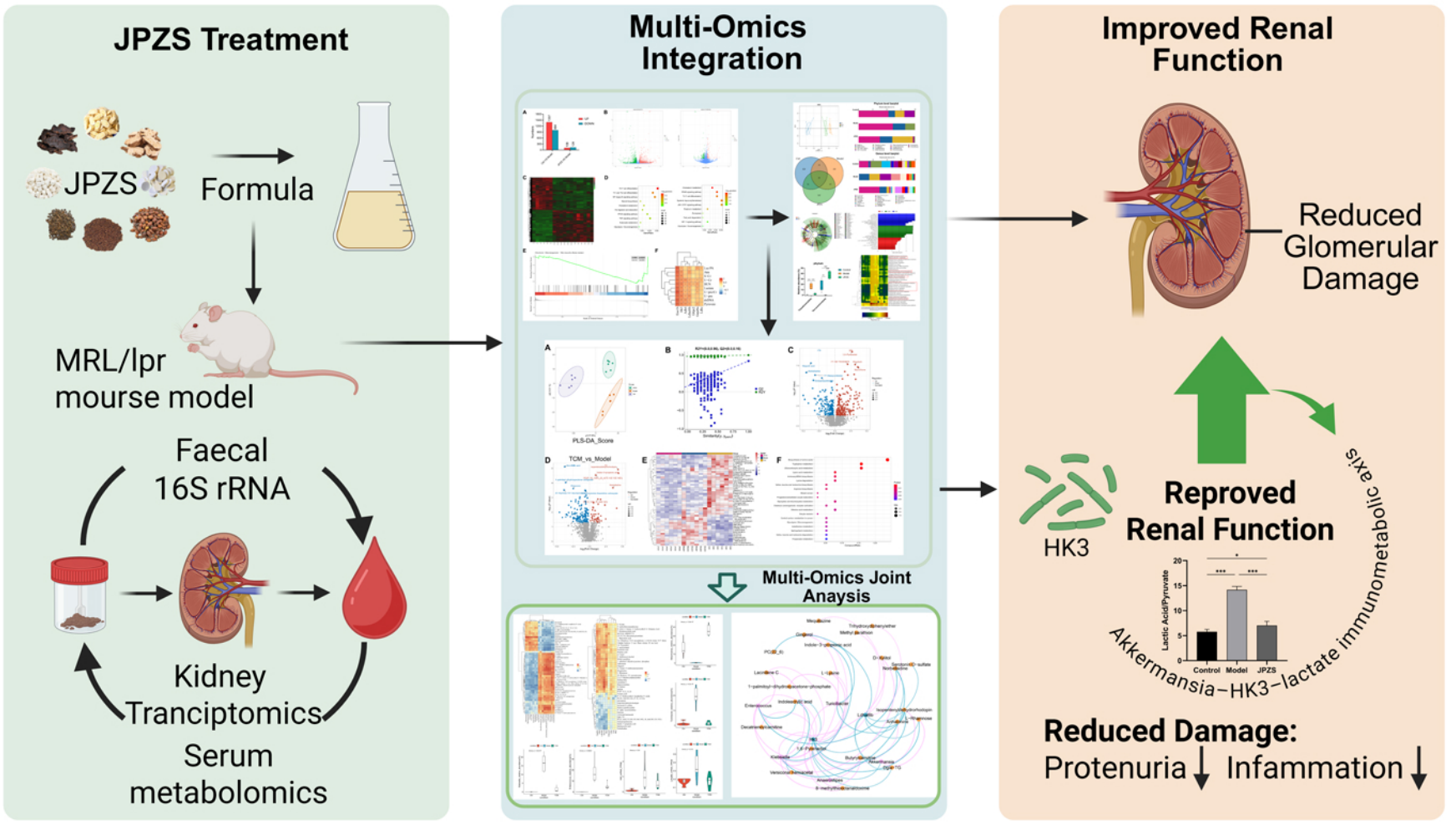

JPZS Treatment
JPZS
Formula
MRL/lpr
mourse model
Faecal
16S rRNA
Kidney
Tranciptomics
Serum
metabolomics
Multi-Omics
Integration
Multi-Omics Joint
Anaysis
Improved Renal
Function
Reduced
Glomerular
Damage
HK3
Reproved
Renal Function
Akkermansia−HK3−lactate immunometabolic axis
Lactic Acid/Pyruvate
Control
Model
JPZS
Reduced Damage:
Protenuria
Infammation
