# Supplementary material for: Multi-omics profiling identifies a putative Akkermansia–HK3–lactate immunometabolic link associated with JPZS treatment in lupus nephritis
Source: Front Immunol. 2026 Oct 22;17:1873518. doi: 10.3389/fimmu.2026.1873518 (PMC13735744; doi:10.3389/fimmu.2026.1873518)
Supplement: Supplementary file 4 [file SupplementaryFile1.docx]

Supplementary Raw Data - Upload Manifest

========================================

Journal: Frontiers in Immunology

Manuscript ID: 1873518

Total ZIP packages: 15

Total size: ~263 MB

INSTRUCTIONS FOR REVIEWERS/EDITORS

------------------------------------

All raw data are organized across 15 ZIP files. Each ZIP corresponds to a

specific data category or figure group. Extract all ZIPs into the same root

folder to restore the original directory structure:

Frontiers_RawData_Submission_1873518/

├── 01_Source_Data_for_Figures_and_Tables/

├── 02_Original_Microscopy_Images/

├── 03_Original_Western_Blot_Full_Scans/

├── 04_Analysis_Scripts/

├── 05_Ethics_Approval/

├── 06_Public_Deposition_and_Working_Sheets/

└── 07_Checklists_and_Reports/

ZIP FILE INDEX

==============

01 - Source Data for Figures and Tables

----------------------------------------

Part1_SourceData_Part1.zip (1.4 MB)

Contents: Figure1 (biochemistry, histology, IF)

Figure3 (16S alpha/beta diversity, taxonomy)

Figure4 (LEfSe, PICRUSt2, taxa)

Part1_SourceData_Part2_Part1.zip (21.1 MB)

Contents: Figure2 RNAseq - gene count matrix, FPKM matrix,

GSEA/KEGG enrichment summaries (first batch)

Part1_SourceData_Part2_Part2.zip (19.8 MB)

Contents: Figure2 RNAseq - mRNA expression summary,

differential expression summary,

GO/KEGG enrichment (second batch)

Part1_SourceData_Part3.zip (12.0 MB)

Contents: Figure5 (serum metabolomics)

Figure6 (multi-omics, qPCR, WB, lactate/pyruvate)

Table1 (GSEA)

02 - Original Microscopy Images

---------------------------------

Part7_IgG_Part1.zip (26.5 MB)

Contents: Fig1G - IgG immunofluorescence images (Part 1 of 2)

Part7_IgG_Part2.zip (19.3 MB)

Contents: Fig1G - IgG immunofluorescence images (Part 2 of 2)

Part8_HE_Part1.zip (29.1 MB)

Contents: Fig1H - HE staining images (Part 1 of 2)

Part8_HE_Part2.zip (16.6 MB)

Contents: Fig1H - HE staining images (Part 2 of 2)

Part9_MASSON_Part1.zip (29.0 MB)

Contents: Fig1H - Masson's trichrome staining images (Part 1 of 2)

Part9_MASSON_Part2.zip (15.6 MB)

Contents: Fig1H - Masson's trichrome staining images (Part 2 of 2)

Part10_PAS_Part1.zip (26.2 MB)

Contents: Fig1H - PAS staining images (Part 1 of 2)

Part10_PAS_Part2.zip (14.8 MB)

Contents: Fig1H - PAS staining images (Part 2 of 2)

03 - Original Western Blot Full Scans

---------------------------------------

Part2_WesternBlot.zip (12.0 MB)

Contents: Fig6J full-scan blots - GAPDH, HK3, LDHAL6B

Lane map and densitometry data

04 - Analysis Scripts

----------------------

Part3_Scripts.zip (14.2 MB)

Contents: All analysis scripts (R / Python / Shell) used for

RNAseq, 16S, metabolomics, and multi-omics integration

05/06/07 - Ethics, Deposition, Checklists

-------------------------------------------

Part4_Ethics_Deposition_Checklists.zip (5.5 MB)

Contents: Ethics approval documentation

GEO / SRA / Metabolomics repository submission receipts

and accession records

Submission checklist and reporting documents

NOTES

------

- Each ZIP was kept under 30 MB to comply with the Frontiers upload size limit.

- Multi-part ZIPs (e.g. Part1/Part2) belong to the same source folder

and should be extracted together to restore the complete directory.
