# Supplementary material for: Multi-omics profiling identifies a putative Akkermansia–HK3–lactate immunometabolic link associated with JPZS treatment in lupus nephritis
Source: Front Immunol. 2026 Oct 22;17:1873518. doi: 10.3389/fimmu.2026.1873518 (PMC13735744; doi:10.3389/fimmu.2026.1873518)
Supplement: Supplementary file 5 [file Table1.docx]

**Supplementary Table S1. Targeted LC–MS/MS parameters for chemical characterisation of JPZS**

**A. Reference standards used for constituent identification**

| **No.** | **Compound** | **Catalogue no.** | **CAS no.** |
| --- | --- | --- | --- |
| 1 | 5-Hydroxymethylfurfural | B21382 | 67-47-0 |
| 2 | Atractylenolide III | B20056 | 73030-71-4 |
| 3 | Atractylone | B20129 | 6989-21-5 |
| 4 | Atractylodin | B20128 | 55290-63-6 |
| 5 | Gallic acid | B20851 | 149-91-7 |
| 6 | Acteoside | B20715 | 61276-17-3 |
| 7 | Catalpol | B21678 | 2415-24-9 |
| 8 | Glucose | B21882 | 50-99-7 |
| 9 | Catechin | B21722 | 154-23-4 |
| 10 | Homovanillic acid | B20433 | 306-08-1 |
| 11 | Hyperoside | B20631 | 482-36-0 |
| 12 | Rutin | B20711 | 153-18-7 |
| 13 | Ellagic acid | B21073 | 476-66-4 |
| 14 | Quercetin | B20257 | 117-39-5 |
| 15 | Tiliroside | B21587 | 20316-62-5 |
| 16 | Kaempferol | B21126 | 520-18-3 |
| 17 | Formononetin | B20836 | 485-72-3 |
| 18 | Astragaloside A | B20564 | 84687-43-4 |
| 19 | Dioscin | B21176 | 19057-60-4 |
| 20 | Pachymic acid | B20400 | 29070-92-6 |

All reference standards were obtained from Shanghai Yuanye Bio-Technology Co., Ltd. (Shanghai, China). Compound identities in the JPZS preparation were assessed by matching retention times and MRM transitions with the corresponding reference standards.

**B. MRM transitions and retention times of representative constituents**

| **No.** | **Compound** | **Ion mode** | **Quantifier transition (m/z)** | **Qualifier transition(s) (m/z)** | **Retention time (min)** |
| --- | --- | --- | --- | --- | --- |
| 1 | 5-Hydroxymethylfurfural | MRM(+) | 127.1000 → 81.1000 | 127.1000 → 109.500 | 1.035 |
| 2 | Atractylenolide III | MRM(+) | 249.1000 → 105.1000 | 249.1000 → 107.100 | 4.223 |
| 3 | Atractylone | MRM(+) | 217.2500 → 95.2000 | 217.2500 → 105.150 | 6.736 |
| 4 | Atractylodin | MRM(+) | 183.1500 → 128.1000 | 183.1500 → 115.0000; 183.1500 → 77.150 | 5.845 |
| 5 | Gallic acid | MRM(−) | 169.1000 → 124.0500 | 169.1000 → 78.9500 | 1.021 |
| 6 | Acteoside | MRM(−) | 623.1500 → 161.1000 | 623.1500 → 461.200 | 1.221 |
| 7 | Catalpol | MRM(−) | 361.1500 → 97.0000 | 361.1500 → 163.100 | 0.841 |
| 8 | Glucose | MRM(−) | 179.1000 → 58.9000 | 179.1000 → 70.900 | 0.793 |
| 9 | Catechin | MRM(−) | 289.1000 → 123.0500 | 289.1000 → 109.050 | 1.007 |
| 10 | Homovanillic acid | MRM(−) | 181.0500 → 121.2000 | 181.0500 → 93.1000 | 1.482 |
| 11 | Hyperoside | MRM(−) | 463.0500 → 300.0500 | 463.0500 → 271.050 | 1.914 |
| 12 | Rutin | MRM(−) | 609.1000 → 300.0000 | 609.1000 → 271.050 | 1.778 |
| 13 | Ellagic acid | MRM(−) | 301.0500 → 145.0500 | 301.0500 → 284.150 | 2.875 |
| 14 | Quercetin | MRM(−) | 301.0000 → 151.0000 | 301.0000 → 121.0000; 301.0000 → 107.0000 | 2.885 |
| 15 | Tiliroside | MRM(−) | 593.1000 → 285.0500 | 593.1000 → 255.0500; 593.1000 → 145.0500 | 2.916 |
| 16 | Kaempferol | MRM(−) | 284.8500 → 159.1000 | 284.8500 → 117.000 | 3.444 |
| 17 | Formononetin | MRM(−) | 267.0500 → 223.0500 | 267.0500 → 251.050 | 3.714 |
| 18 | Astragaloside A | MRM(−) | 829.2500 → 783.4000 | 829.2500 → 118.900 | 4.901 |
| 19 | Dioscin | MRM(−) | 913.4000 → 721.4000 | 913.4000 → 205.500 | 5.770 |
| 20 | Pachymic acid | MRM(−) | 527.3500 → 465.3000 | 527.3500 → 405.350 | 6.115 |

**C. Detailed MRM acquisition parameters**

| **Compound** | **Ion mode** | **Precursor ion (m/z)** | **Product ion (m/z)** | **Dwell time (ms)** | **Q1 bias (V)** | **Collision energy (V)** | **Q3 bias (V)** | **Retention time (min)** |
| --- | --- | --- | --- | --- | --- | --- | --- | --- |
| 5-Hydroxymethylfurfural | MRM(+) | 127.1 | 109.5 | 10 | -10 | -15 | -22 | 1.035 |
| 5-Hydroxymethylfurfural | MRM(+) | 127.1 | 81.1 | 10 | -10 | -19 | -19 | 1.035 |
| Atractylenolide III | MRM(+) | 249.1 | 105.1 | 10 | -12 | -35 | -25 | 4.223 |
| Atractylenolide III | MRM(+) | 249.1 | 107.1 | 10 | -17 | -28 | -23 | 4.223 |
| Atractylone | MRM(+) | 217.25 | 95.2 | 10 | -15 | -23 | -21 | 6.736 |
| Atractylone | MRM(+) | 217.25 | 105.15 | 10 | -16 | -29 | -21 | 6.736 |
| Atractylodin | MRM(+) | 183.15 | 128.1 | 10 | -22 | -23 | -22 | 5.845 |
| Atractylodin | MRM(+) | 183.15 | 115 | 10 | -21 | -19 | -22 | 5.845 |
| Atractylodin | MRM(+) | 183.15 | 77.15 | 10 | -14 | -29 | -15 | 5.845 |
| Gallic acid | MRM(−) | 169.1 | 78.95 | 10 | 19 | 23 | 23 | 1.021 |
| Gallic acid | MRM(−) | 169.1 | 124.05 | 10 | 20 | 20 | 23 | 1.021 |
| Acteoside | MRM(−) | 623.15 | 161.1 | 10 | 32 | 35 | 15 | 1.221 |
| Acteoside | MRM(−) | 623.15 | 461.2 | 10 | 24 | 28 | 21 | 1.221 |
| Catalpol | MRM(−) | 361.15 | 97 | 10 | 24 | 25 | 17 | 0.841 |
| Catalpol | MRM(−) | 361.15 | 163.1 | 10 | 13 | 15 | 30 | 0.841 |
| Glucose | MRM(−) | 179.1 | 58.9 | 10 | 19 | 19 | 22 | 0.793 |
| Glucose | MRM(−) | 179.1 | 70.9 | 10 | 21 | 16 | 23 | 0.793 |
| Catechin | MRM(−) | 289.1 | 109.05 | 10 | 16 | 25 | 16 | 1.007 |
| Catechin | MRM(−) | 289.1 | 123.05 | 10 | 20 | 30 | 20 | 1.007 |
| Homovanillic acid | MRM(−) | 181.05 | 121.2 | 10 | 20 | 31 | 10 | 1.482 |
| Homovanillic acid | MRM(−) | 181.05 | 93.1 | 10 | 20 | 42 | 14 | 1.482 |
| Hyperoside | MRM(−) | 463.05 | 300.05 | 10 | 16 | 28 | 29 | 1.914 |
| Hyperoside | MRM(−) | 463.05 | 271.05 | 10 | 23 | 47 | 26 | 1.914 |
| Rutin | MRM(−) | 609.1 | 300 | 10 | 32 | 38 | 28 | 1.778 |
| Rutin | MRM(−) | 609.1 | 271.05 | 10 | 32 | 54 | 28 | 1.778 |
| Ellagic acid | MRM(−) | 301.05 | 284.15 | 10 | 14 | 33 | 11 | 2.875 |
| Ellagic acid | MRM(−) | 301.05 | 145.05 | 10 | 21 | 35 | 25 | 2.875 |
| Quercetin | MRM(−) | 301 | 151 | 10 | 22 | 23 | 12 | 2.885 |
| Quercetin | MRM(−) | 301 | 121 | 10 | 21 | 25 | 22 | 2.885 |
| Quercetin | MRM(−) | 301 | 107 | 10 | 15 | 28 | 15 | 2.885 |
| Tiliroside | MRM(−) | 593.1 | 285.05 | 10 | 30 | 35 | 27 | 2.916 |
| Tiliroside | MRM(−) | 593.1 | 255.05 | 10 | 28 | 55 | 21 | 2.916 |
| Tiliroside | MRM(−) | 593.1 | 145.05 | 10 | 22 | 40 | 22 | 2.916 |
| Kaempferol | MRM(−) | 284.85 | 159.1 | 10 | 21 | 30 | 25 | 3.444 |
| Kaempferol | MRM(−) | 284.85 | 117 | 10 | 30 | 42 | 17 | 3.444 |
| Formononetin | MRM(−) | 267.05 | 223.05 | 10 | 13 | 32 | 21 | 3.714 |
| Formononetin | MRM(−) | 267.05 | 251.05 | 10 | 13 | 30 | 25 | 3.714 |
| Astragaloside A | MRM(−) | 829.25 | 783.4 | 10 | 24 | 27 | 26 | 4.901 |
| Astragaloside A | MRM(−) | 829.25 | 118.9 | 10 | 24 | 41 | 10 | 4.901 |
| Dioscin | MRM(−) | 913.4 | 721.4 | 10 | 34 | 38 | 22 | 5.770 |
| Dioscin | MRM(−) | 913.4 | 205.5 | 10 | 26 | 50 | 18 | 5.770 |
| Pachymic acid | MRM(−) | 527.35 | 465.3 | 10 | 28 | 38 | 28 | 6.115 |
| Pachymic acid | MRM(−) | 527.35 | 405.35 | 10 | 26 | 50 | 25 | 6.115 |

Q1 bias, collision energy, and Q3 bias values are reported using the sign convention recorded in the LCMS-8045 acquisition method.

**D. LC–MS/MS conditions**

| **Parameter** | **Value** |
| --- | --- |
| LC instrument | LC-40A |
| Column | Shim-pack GIST C18, 2 μm, 2 × 100 mm |
| Mobile phase A | Water containing 0.01% ammonium formate |
| Mobile phase B | Methanol containing 0.01% formic acid |
| Flow rate | 0.3 mL/min |
| Injection volume | 5 μL |
| Column temperature | 40 °C |
| Gradient program | 0 min: 50% B; 5 min: 100% B; 8 min: 100% B; 8.1 min: 50% B |
| Total run time | 14 min |
| MS instrument | LCMS-8045 |
| Ion source | Electrospray ionisation (ESI) |
| Ion modes | Positive and negative ion modes |
| Nebulizing gas | Nitrogen, 3 L/min |
| Drying gas | Nitrogen, 10 L/min |
| Collision gas | High-purity argon |
| Desolvation line temperature | 526 °C |
| Heat block temperature | 300 °C |
| Acquisition mode | Multiple reaction monitoring (MRM) |
| Dwell time | 10 ms |

Abbreviations: ESI, electrospray ionisation; JPZS, Qihuang Jianpi Zishen decoction; MRM, multiple reaction monitoring; m/z, mass-to-charge ratio.
