# Supplementary material for: Multi-omics profiling identifies a putative Akkermansia–HK3–lactate immunometabolic link associated with JPZS treatment in lupus nephritis
Source: Front Immunol. 2026 Oct 22;17:1873518. doi: 10.3389/fimmu.2026.1873518 (PMC13735744; doi:10.3389/fimmu.2026.1873518)
Supplement: Supplementary file 6 [file Table2.docx]

**Supplementary Table S2. Primer sequences used for quantitative real-time PCR**

| **Gene** | **Amplicon Size (bp)** | **Forward Primer (5′→3′)** | **Reverse Primer (5′→3′)** |
| --- | --- | --- | --- |
| β-actin | 120 | AGTGTGACGTTGACATCCGT | TGCTAGGAGCCAGAGCAGTA |
| Hk3 | 123 | TGTTGCTTGTCGCCTTAC | GGTTTCTTTGGGAGTTGTG |
| Ldhal6b | 101 | TGTTCACTTCCTCCACCT | AAGCCCTTGAAATCTACC |

Abbreviations: bp, base pairs.
